# Bioinformatic Analysis of Prognostic and Immune-Related Genes in Pancreatic Cancer

**DOI:** 10.1155/2021/5549298

**Published:** 2021-08-03

**Authors:** Ziang Li, Chang Hu, Zhiqiang Yang, Minlan Yang, Jiayu Fang, Xuhong Zhou

**Affiliations:** ^1^Department of Gastroenterology, Zhongnan Hospital of Wuhan University, 65 Donghu Road, Wuhan, Hubei 430071, China; ^2^Department of Intensive Care Unit, Zhongnan Hospital of Wuhan University, 65 Donghu Road, Wuhan, Hubei 430071, China; ^3^Department of Spine Surgery and Musculoskeletal Tumor, Zhongnan Hospital of Wuhan University, 65 Donghu Road, Wuhan, Hubei 430071, China; ^4^Department of Otorhinolaryngology-Head and Neck Surgery, Zhongnan Hospital of Wuhan University, 65 Donghu Road, Wuhan, Hubei 430071, China

## Abstract

Pancreatic cancer (PC) is a malignant tumor with poor prognosis. The poor effect of surgery and chemotherapy makes the research of immunotherapy target molecules significant. Therefore, identifying the new molecular targets of PC is important for patients. In our study, we systematically analyzed molecular correlates of pancreatic cancer by bioinformatic analysis. We characterized differentially expressed analysis based on the TCGA pancreatic cancer dataset. Then, univariate Cox regression was employed to screen out overall survival- (OS-) related DEGs. Based on these genes, we established a risk signature by the multivariate Cox regression model. The ICGC cohort and GSE62452 cohort were used to validate the reliability of the risk signature. The impact of T lymphocyte-related genes from risk signature was confirmed in PC. Here, we observed the correlation between the T lymphocyte-related genes and the expression level of targeted therapy. We established a five-mRNA (LY6D, ANLN, ZNF488, MYEOV, and SCN11A) prognostic risk signature. Next, we identified ANLN and MYEOV that were associated with T lymphocyte infiltrations (*P* < 0.05). High ANLN and MYEOV expression levels had a poorer prognosis in decreased T lymphocyte subgroup in PC. Correlation analysis between ANLN and MYEOV and immunomodulators showed that ANLN and MYEOV may have potential value in pancreatic cancer immunotherapy.

## 1. Introduction

Pancreatic cancer is a malignant tumor with fewer than 7% of patients surviving the past 5 years [1]. It has one of the worst outcomes among all cancers with a median survival of approximately 6 months [2]. Pancreatic cancer is forecast to be the second most common cancer in all malignant cancers by 2030 [3]. The high mortality is due to extensive metastasis in the early stages and resistance to therapy. Common chemotherapy options for pancreatic ductal adenocarcinoma (PDAC) such as 5-fluorouracil (5FU), nab-paclitaxel, oxaliplatin, or combination therapy like FOLFIRINOX may lead to tumor resistance [4]. With the prevalence of chemotherapy resistance, immunotherapy may be an emerging treatment of pancreatic cancer. An important component of immunotherapy is cytotoxic T lymphocytes, which can kill cancer cells through antigen-antibody binding, currently known as immunotherapy such as immunomodulators IL-2 or chimeric antigen receptor (CAR) T cell therapy [5, 6]. However, the effect of recent immune therapy trials was not ideal such as checkpoint blockade or engineered T cells, because of the low degree of T cell infiltration in pancreatic cancer [7]. Therefore, our research aims to find molecules, related to clinical significance, T lymphocyte infiltrations, and immune cells of pancreatic cancer patients and explore mechanisms further to serve as potential immunotherapy targets.

## 2. Materials and Methods

### 2.1. Data Acquisition

RNA-seq count files and clinical information of pancreatic cancer (PC) were downloaded from the TCGA website (https://portal.gdc.cancer.gov/) [8] and ICGC website (https://daco.icgc.org/) [9]. GSE62452 data was obtained from the Gene Expression Omnibus (https://www.ncbi.nlm.nih.gov/geo/) [10]. The inclusion criteria were as follows: (a) patients with PC; and(b) complete gene expression profiles and survival information. Finally, 178 PC patients (training cohort) were selected in this study. 66 and 143 PC patients (validation cohorts) were selected, respectively. Immunohistochemical (IHC) sections of genes are publicly available on Human Protein Atlas (https://www.proteinatlas.org/) [11].

### 2.2. Construction and Validation of Prognostic Risk Signature

The “limma” package in *R* software (version 4.0.2) was used to read, normalize, and explore the datasets to identify differentially expressed genes (DEGs) [12]. Univariate Cox regression was performed to screen DEGs significantly associated with overall survival (OS) in the TCGA pancreatic cancer dataset. The Least Absolute Shrinkage and Selection Operator (LASSO) regression was used to reduce data size and select the optimal mRNAs [13]. Multivariable Cox regression was conducted to establish the prognostic risk signature based on the results of LASSO regression. The following formula was used to calculate risk score of each patient: risk score = ∑Ex × C, where *C* is the coefficient, and Ex is the relative expression level of each DEG. The median risk score was used as our cutoff value to divide the PC patients into high-risk and low-risk groups. In addition, Kaplan-Meier survival plot was utilized to analyze the overall survival (OS) difference between the two groups. Second, we evaluate the performance of our model based on ICGC cohort and GSE62452 cohort.

Univariate and multivariate Cox regression analyses were conducted to whether risk score is an independent risk factor of OS in PC patients. Covariates included age, gender, race, stage, T, N, grade, and risk score. The nomogram was formulated to provide visualized risk prediction. The calibration was generated to assess the consistency between actual and predicted survival.

### 2.3. Exploration of Gene-T Lymphocyte Infiltrations Relationships

We calculated the correlation between the gene expression level and GZMB/CD8A ratio. Genes were considered to be related to the T lymphocyte infiltrations when *P* < 0.05 and further discussed relationships with immune cell lines. We obtained gene sets of immune cells from a previous study [14] (Table S1). We computed GSVA (gene set variation analysis) scores by *R*−package “Genome Set Variance Analysis (GSVA).” The top and bottom 25% of GSVA score patients were divided in high and low groups, respectively [15]. By comparing mRNA levels of selected genes in the two groups, we explored the gene-T lymphocyte infiltrations relationships.

### 2.4. The Analysis of Survival Prognosis Based on Gene-T Lymphocyte Infiltrations

The Kaplan-Meier database (http://kmplot.com/analysis/) can analyze the correlation between selected genes and specific tumor prognosis from multiple dimensions [16]. Sources for the databases include Gene Expression Omnibus (GEO), European Genome-phenome Archive (EGA), and The Cancer Genome Atlas (TCGA). Therefore, we used this database to analyze the impact of T lymphocyte cells on survival rates in pancreatic cancer. The hazard ratios (HRs) with 95% confidence intervals (CI) and log-rank *P* values were also calculated.

### 2.5. Gene Set Enrichment Analysis (GSEA)

In this study, we performed single-gene GSEA to explore the potential roles of selected genes included in our risk signature in PC. GSEA generated an initial list on the classification of the genes according to their correlation with each selected gene expression by using the Pearson method. GSEA was performed using GSEA3.0 (http://www.broad.mit.edu/gsea/) [17]. The phenotype label that we put forth was the expression level of the selected gene. The nominal *P* < 0.05 and the FDR < 0.25 were considered statistically significant.

### 2.6. Exploration of the Association between Genes and Immunomodulators

The TISIDB database (http://cis.hku.hk/TISIDB) enables an investigation of the correlations for selected genes with immunomodulators and chemokines [18]. In this study, we used the TISIDB database to investigate correlations with selected genes and immunomodulators.

### 2.7. Statistical Analysis

Statistical analysis was performed with *R* software (version 4.0.2). The “ggplot2” package in *R* was used to draw the volcano plot and heatmaps. The chi-square test was used to evaluate the differences of clinicopathological parameters between the high-risk and low-risk groups. Survival curves were generated by the Kaplan-Meier method. Univariate, LASSO, and multivariate regression analyses were performed to explore the prognostic risk model. Time-dependent ROC analysis was used to evaluate the accuracy of the models. GSVA was performed to compute the immune cell type scores. The results were considered to be statistically significant for *P* < 0.05.

## 3. Results

### 3.1. Differentially Expressed Genes in Cancer Tissues and Normal Tissues

**Figure 1:**
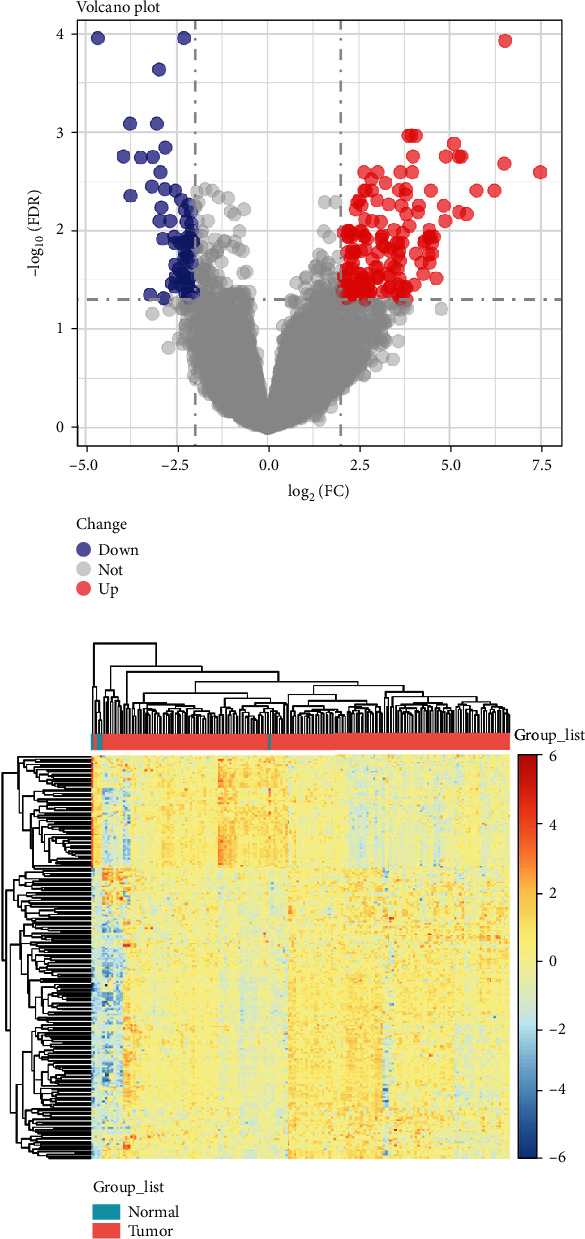
The significantly altered mRNAs in PC samples. (a) Volcano plot revealed the significantly differentially expressed mRNAs between PC and non-PC controls. (b) The differentially expressed mRNAs from TCGA PC cohort were displayed by heat map. PC: pancreatic cancer.

The detailed workflow of our study is shown as a chart (Figure S1). RNA-seq from 186 tumor tissue samples and 36 nontumor samples was downloaded from TCGA. Volcano plot was used to visualize the altered mRNA expression pattern of TCGA PC cohort. A total of 234 DEmRNAs were identified across all the datasets consisting of 168 upregulated and 66 downregulated DEmRNAs (Figure 1(a)). Among them, MUC2 and STAB2 were the most upregulated and downregulated mRNAs in Table S2. Heatmap was used to visualize the expression levels of the significantly differentially expressed mRNAs. The blue color was assigned to the relatively low expression, while the red color represented the relatively high expression (Figure 1(b)).

### 3.2. The Construction and Validation of Prognostic Signature Based on the PC Cohorts

**Figure 2:**
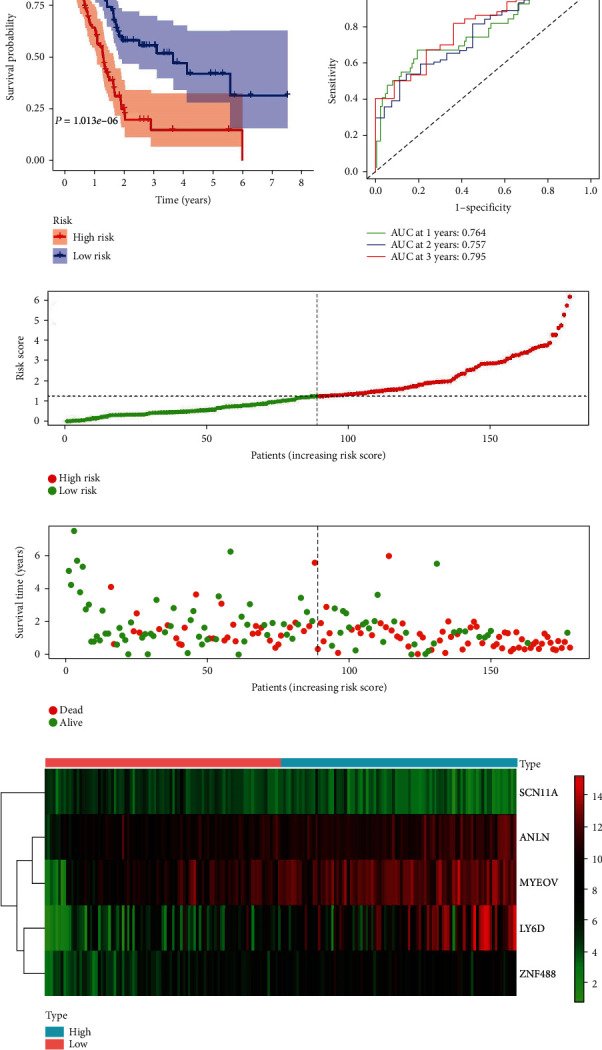
Construction of the prognostic signature based on the TCGA cohort. (a) The OS of patients in the high-risk group shorter than those in the low-risk group. (b) The ROC analysis in the TCGA cohort. (c) The low and high score group for the prognostic signature in PC patients. (d) The survival status and duration of PC patients. (e) Heatmap of the 5 key mRNA expressions in PC. PC: pancreatic cancer.

The mRNAs that were significantly associated with OS were identified by univariate analysis based on TCGA cohort (Table S3). The LASSO regression identified 8 optimal mRNAs including LY6D, FAM83A, ANLN, LAMA3, ZNF488, MYEOV, PLAAT2, and SCN11A (Figure S2). Then, the above eight mRNAs were further subjected to multivariate Cox regression analysis. The multivariate analysis showed that LY6D, ANLN, ZNF488, MYEOV, and SCN11A were the independent prognostic mRNAs for PC. The risk score for each patient was calculated with the following formula: (0.1041 × LY6D) + (0.2339 × ANLN) + (0.1388 × ZNF488) + (0.1260 × MYEOV) + (−0.2197 × SCN11A). Based on the median value of the risk scores, 89 and 88 patients were classified into the high- and low-risk groups, respectively. The OS was significantly shorter in the high-risk group than in the low-risk group (*P* = 1.013*e* − 06) (Figure 2(a)). Then, a time-dependent ROC curve was constructed to determine the predictive accuracy of the prognostic signature. The area under the curve (AUC) of the prognostic signature for 1-year OS, 2-year OS, and 3-year OS was 0.764, 0.757, and 0.795, indicating good predictive accuracy (Figure 2(b)).  Figure 2(c) shows the distributions of risk scores, and the distributions of OS and OS status are demonstrated in Figure 2(d). The expression pattern of these five prognostic mRNAs between high-risk and low-risk groups is shown in Figure 2(e).

To confirm that the prognostic signature that had similar predictive values in different populations, we then used it to predict OS in two independent external validation cohorts using the median risk score as the cutoff.

**Figure 3:**
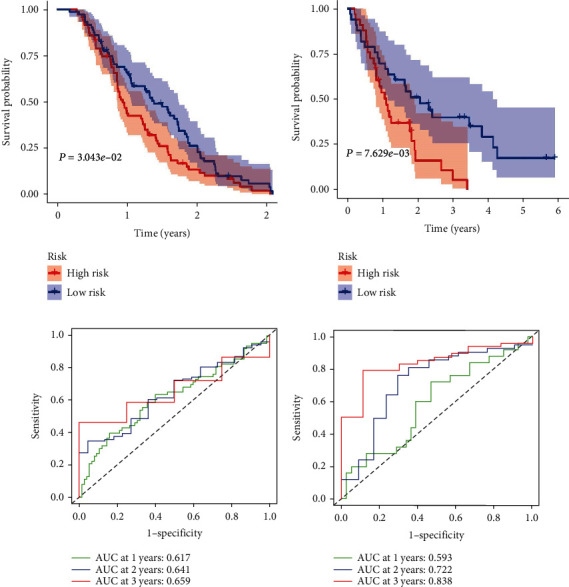
Validation of the prognostic signature with the ICGC and GSE62452 validation cohort. (a) Kaplan-Meier curves of OS in the high-risk and the low-risk groups stratified by the prognostic signature in the ICGC cohort. (b) Kaplan-Meier curves of OS in the high-risk and the low-risk groups stratified by the prognostic signature in the GSE62452. (c) The ROC analysis in the ICGC cohort. (d) The ROC analysis in the GSE62452.

A total of 143 patients in the ICGC cohort (validation cohort -1) were classified into a low-risk group (*n* = 72) and a high-risk group (*n* = 71), and the OS of the PC patients in the high-risk group was significantly lower than that of the patients in the low-risk group (*P* = 3.043*e* − 02; Figure 3(a)). The prognostic signature constructed with the ICGC cohort also showed a favorable predictive ability for the 1-, 2-, and 3-year OS rates, with AUC values of 0.617, 0.641, and 0.659, respectively (Figure 3(c)). In addition, as shown in Figure 3(b), a total of 66 patients in the GEO cohort GSE62452 (validation cohort-2) were classified into a low-risk group (*n* = 33) and a high-risk group (*n* = 33), and the OS of the PC patients in the high-risk group was significantly lower than that of patients in the low-risk group (*P* = 7.629*e* − 03). The result generated by the GSE62452 cohort also showed a favorable predictive ability for the 1-, 2-, and 3-year OS rates, with AUC values of 0.593.0.722, and 0.833, respectively (Figure 3(d)).

**Figure 4:**
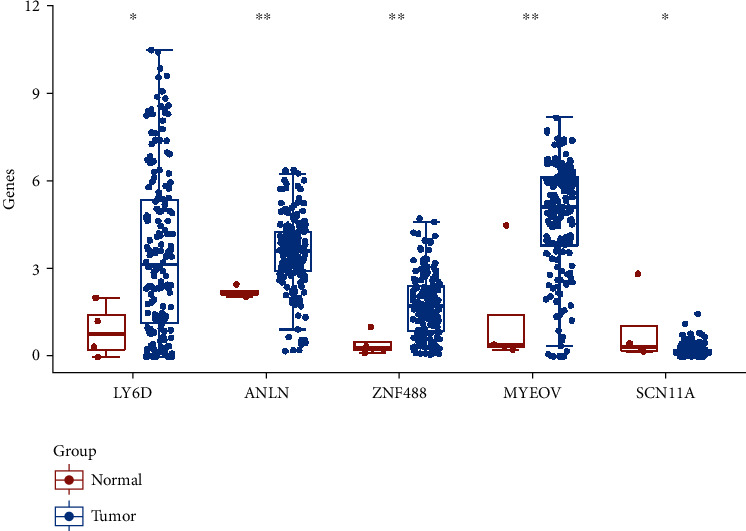
The expression distribution of LY6D, ANLN, ZNF488, MYEOV, and SCN11A in tumor tissues and normal tissues. Asterisks represent levels of significance. ∗*P* < 0.05, ∗∗*P* < 0.01, ∗∗∗*P* < 0.001.

Furthermore, we analyzed the expression levels of LY6D, ANLN, ZNF488, MYEOV, and SCN11A in PC tissues. We found that these five genes are highly expressed in tumor tissues (Figure 4).

**Figure 5:**
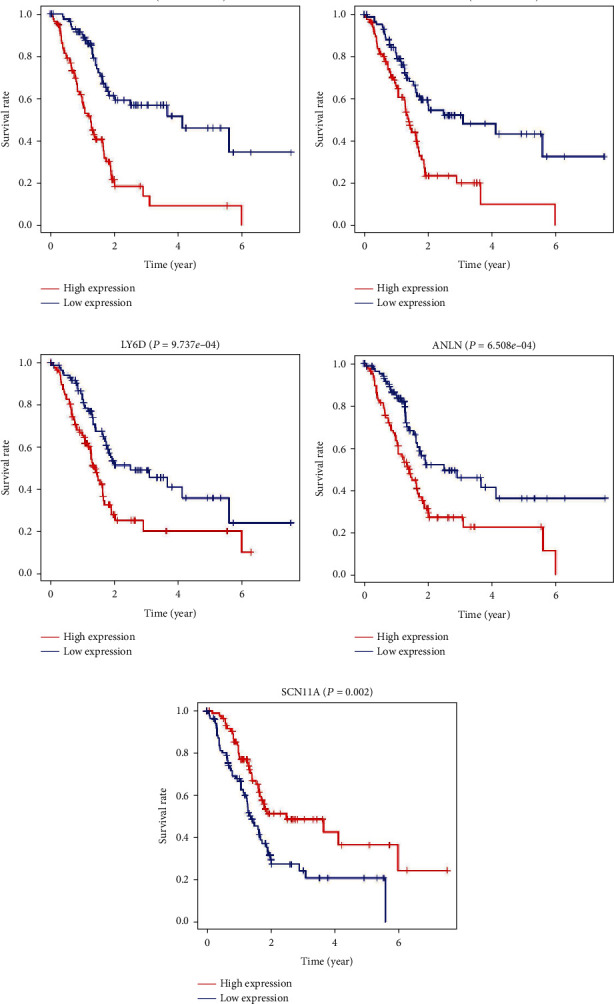
Kaplan-Meier survival curves for the 5 prognostic mRNAs for PC in the TCGA dataset. MYEOV, LY6D, ANLN, and ZNF488 were unfavorable factors, and SCN11A was confirmed to be favorable prognostic factors for PC. PC: pancreatic cancer.

Among the 5 genes in the OS-related prediction model, the high expression of MYEOV (*P* = 2.336*e* − 08), LY6D (*P* = 9.737*e* − 04), ANLN (*P* = 6.508*e* − 04), and ZNF488 (*P* = 1.366*e* − 04) genes was associated with worse prognosis in PC in Kaplan–Meier curves according to the median values of the gene expression (Figures 5(a)–5(d)). In addition, the high expression of the SCN11A (*P* = 9.737*e* − 04) gene was associated with better prognosis in Kaplan–Meier curves according to the median values of the gene expression (Figure 5(e)).

**Figure 6:**
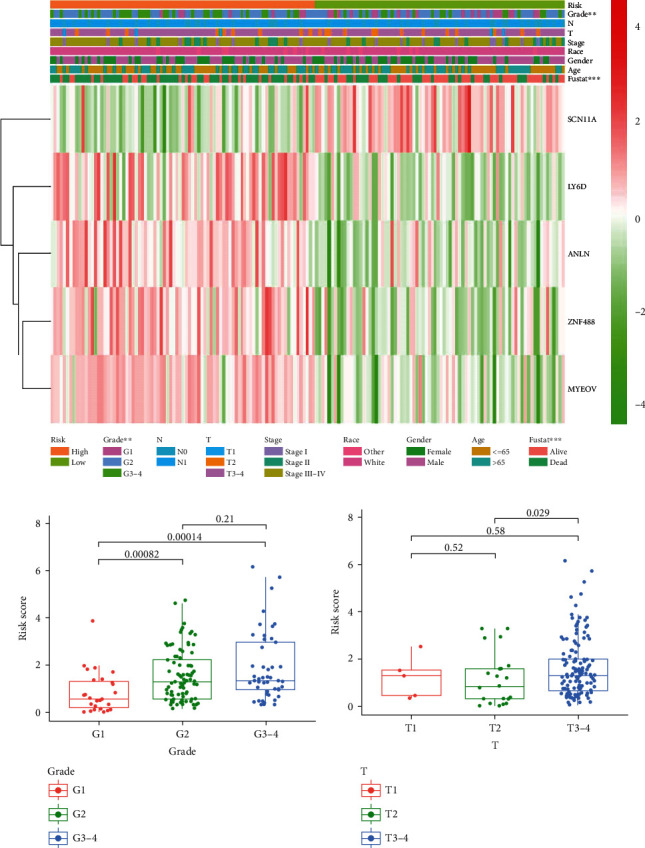
Correlation with risk score and clinical traits in TCGA Cohort. (a) Heatmap and clinicopathologic features of high- and low-risk groups. (b, c) Distribution of risk scores stratified by grade (b) and tumor stage (c). ∗*P* < 0.05, ∗∗*P* < 0.01, and ∗∗∗*P* < 0.001.

Furthermore, the correlation analysis between the risk group and clinicopathologic features find that high risk score is closely related to tumor grade (G1 vs G2 *P* = 0.00082, G1 vs G3-4 *P* = 0.00014) and T stage (T2 vs T3-4, *P* = 0.029), but is not related to gender, race, age, pathological stage, and N stage (Figure 6, Figure S3).

### 3.3. The Prognostic Signature Is an Independent Prognostic Factor for Pancreatic Patients by Cox Regression Analyses

**Figure 7:**
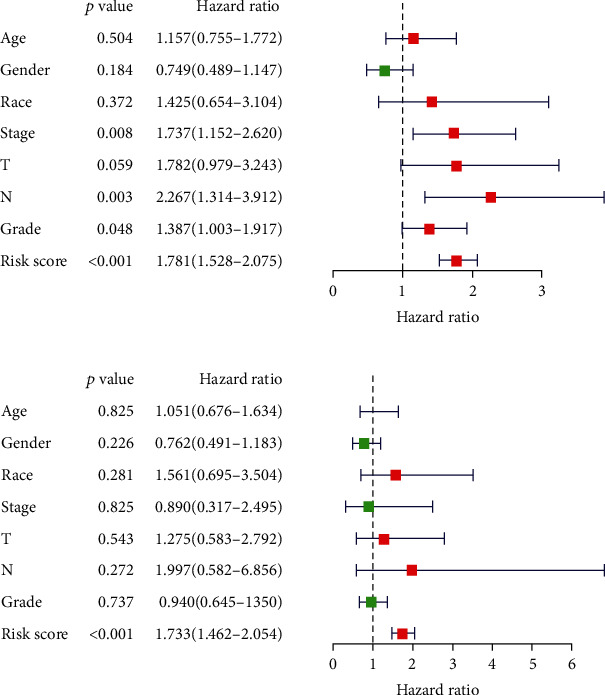
Results of Cox regression for risk factors for PC. (a) Result of univariate Cox regression. (b) Result of multivariate Cox regression. PC: pancreatic cancer.

To determine whether the prognostic signature for OS is an independent prognostic factor for PC patients, we performed Cox regression analysis. Univariate Cox regression analysis showed that stage, grade, T stage, N stage, and risk score were significantly associated with OS in PC patients (Figure 7(a)). Multivariate Cox regression analysis showed that risk score was an independent factor influencing PC prognosis (Figure 7(b)).

### 3.4. Nomogram Model Construction and Prediction

To establish a clinically applicable method for predicting the prognosis of PC patients, we established a prognostic nomogram to predict the survival probability at 1, 2, and 3 years based on the TCGA cohort.

**Figure 8:**
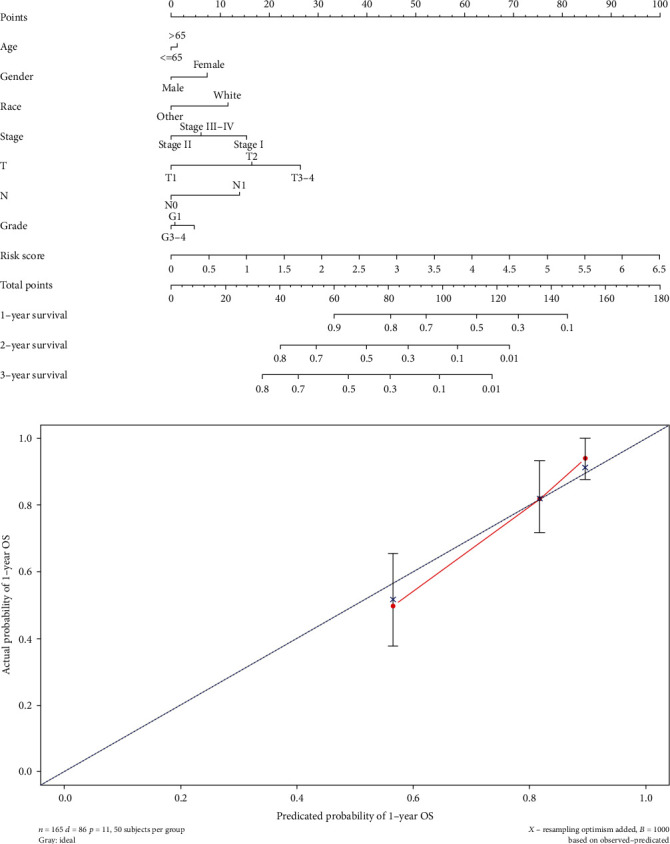
Construction of a robust nomogram prediction model. (a) The nomogram prediction model was built up using the risk signature and other clinicopathological parameters. (b) The calibration plots demonstrated that the nomogram model showed excellent performance for predicting the 1-year OS. OS: overall survival.

As shown in Figure 7(a), the risk signature and other clinicopathological parameters such as age, gender, race, tumor grade, stage, T stage, and N stage were included in the nomogram model to predict the prognosis of PC. A nomogram-based score for each patient was obtained according to the risk score and clinical parameters on the point scale. The 1-year OS, 2-year OS, or 3-year OS of each PC patient could be predicted by calculating the total nomogram score. The calibration curves showed that the nomogram model we built up exhibited good performance for predicting the 1-year OS of PC (Figure 8(b)).

### 3.5. The Impact of T Lymphocyte-Related Genes in Pancreatic Cancer

**Figure 9:**
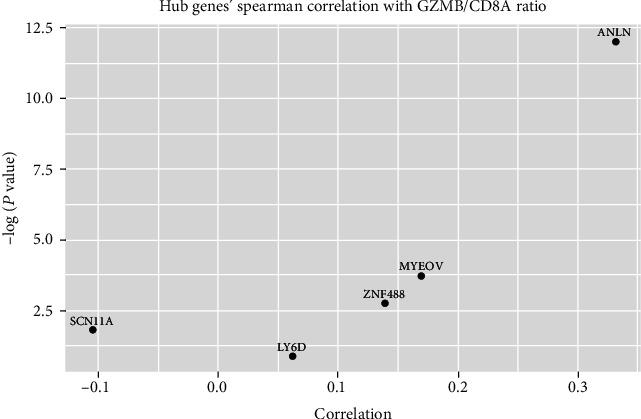
Scatter diagram of 5 screened genes and mRNA level Spearman correlation with the GZMB/CD8A ratio.

**Figure 10:**
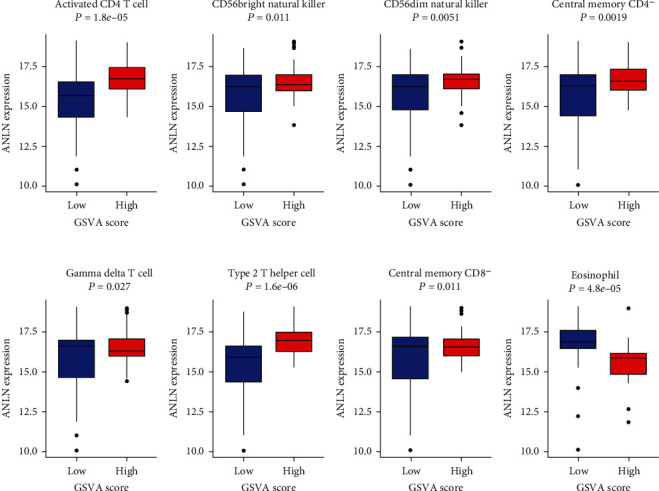
ANLN expression in 8 immune cell lines in two GSVA score groups.

**Figure 11:**
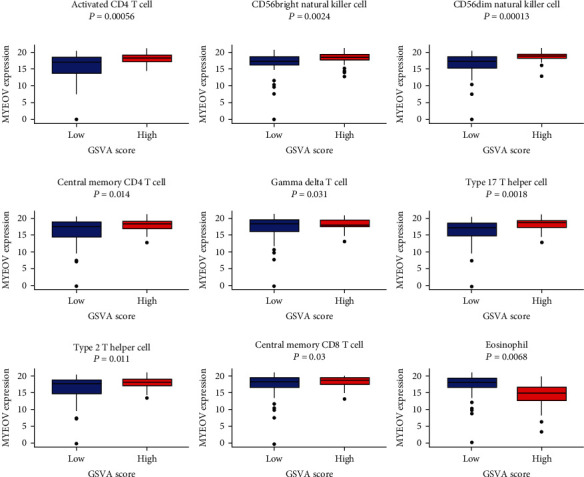
MYEOV expression in 9 immune cell lines in two GSVA score groups.

We have confirmed that the expressions of ANLN (*P* = 5.98*e* − 06, correlation score = 0.33108779) and MYEOV (*P* = 0.023, correlation score = 0.16896960) were associated with the GZMB/CD8A ratio (Figure 9). ANLN was observed to be related to 8 immune cell lines, and MYEOV was related to 9 immune cell lines (Figures 10 and 11).

**Figure 12:**
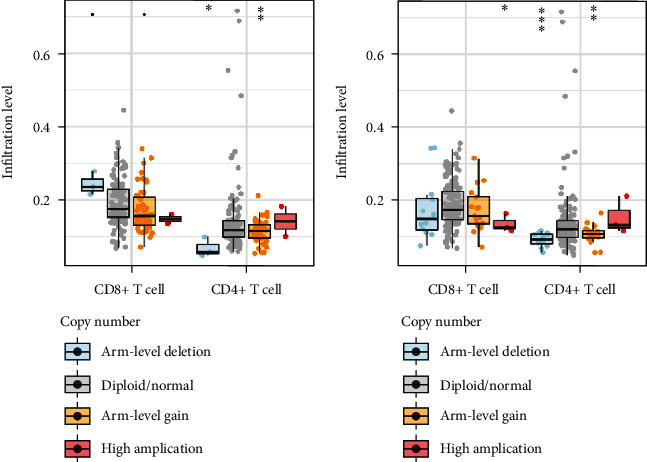
Effect of the genetic alterations of ANLN and MYEOV on the T immune cell infiltration. (a) ANLN. (b) MYEOV. ∗*P* < 0.05, ∗∗*P* < 0.01, and ∗∗∗*P* < 0.001.

To elucidate the underlying mechanisms by which the ANLN and MYEOV were associated with different T cell immune infiltrations, we analyzed the effects of somatic cell copy number alternations (CNAs) of the ANLN and MYEOV on T immune cell infiltration. The CNAs of the identified ANLN and MYEOV, including arm-level deletion and arm-level gain, significantly affected the infiltration level of CD4+ T cells (Figure 12).

**Figure 13:**
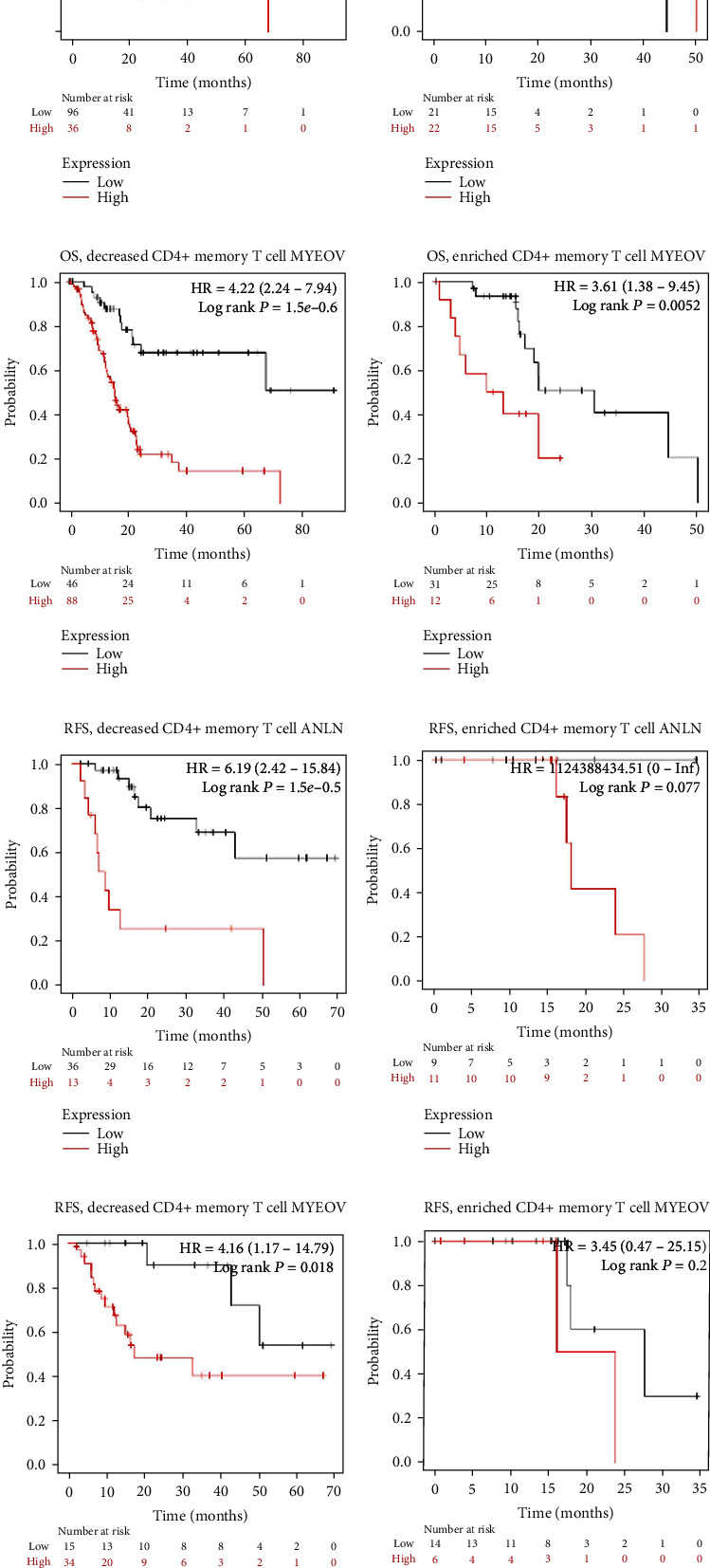
Comparison of Kaplan-Meier survival curves of the high and low expression of ANLN and MYEOV in PC based on immune cells subgroups.

The results showed that the expression of ANLN of PC in decreased CD4+ memory T cells cohort had poorer OS and RFS, respectively (OS, log rank *P* = 3.4*e* − 05; RFS, log rank *P* = 1.5*e* − 05) (Figures 13(a) and 13(e)), But there was no significant correlation between the high ANLN and the prognosis of OS or RFS in the enriched CD4+ memory T cells (OS, log rank *P* = 0.32; RFS, log rank *P* = 0.077), respectively (Figures 13(b) and 13(f)). For the high MYEOV, there was only no significant correlation between the high MYEOV and the prognosis of RFS in the enriched CD4+ memory T cells (RFS, log rank *P* = 0.2) (Figure 13(h)).

### 3.6. Underlying Mechanisms of the ANLN and MYEOV in PC

**Figure 14:**
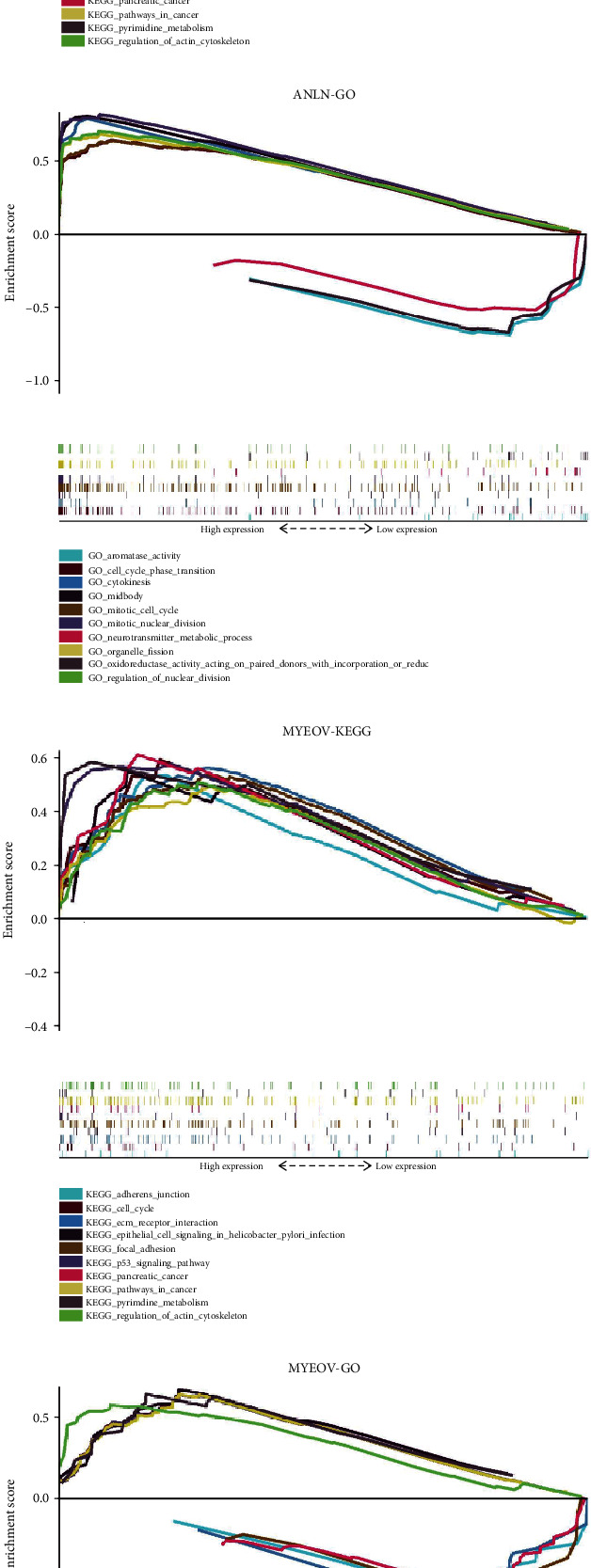
Gene set enrichment analysis results based on ANLN and MYEOV. (a, b) shows the results of ANLN and MYEOV, respectively.

Interestingly, we found that the expression levels of ANLN and MYEOV have a significant positive correlation (Figure S4). Then, we analyzed the expression of the proteins encoded by the two genes using clinical specimens from the Human Protein Profiles. ANLN and MYEOV were moderately positive in the PC tissue relative to their expression levels in the normal tissue (Figure S5). In addition, the single-gene GSEA results show that the two genes have many of the same significantly enriched KEGG pathways (Figure 14).

### 3.7. Effectiveness Predicting of Targeted Immunomodulators with ANLN and MYEOV

Immunomodulators can be further classified into immunoinhibitors, immunostimulators and major histocompatibility complex (MHC) molecules.

**Figure 15:**
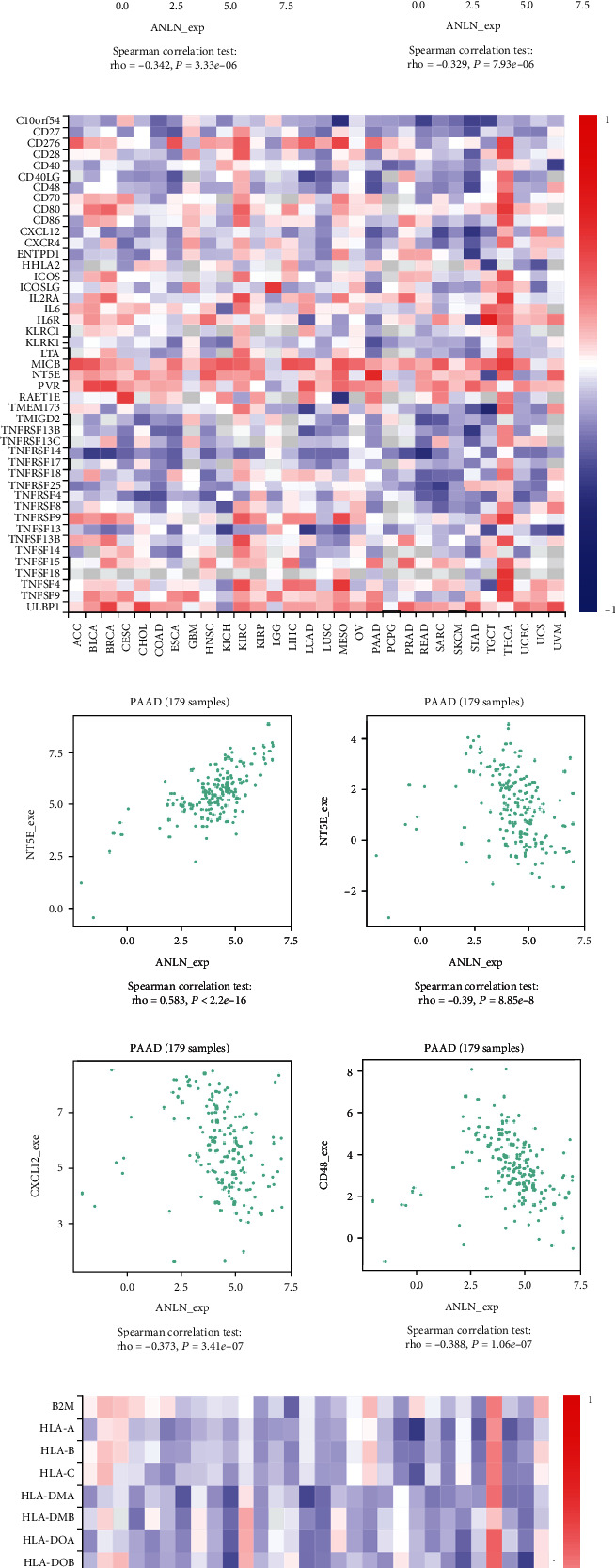
Spearman's correlation of ANLN with immunomodulators. (A) Relations between the immunoinhibitors and ANLN expression. (B) Top 4 immunoinhibitors displaying the greatest Spearman's correlation with ANLN expression. (C) Relations between immunostimulators and ANLN expression. (D) Top 4 immunostimulators displaying the greatest Spearman's correlation with ANLN expression. (E) Relations between MHC molecules and ANLN expression. (F) Top 4 MHC molecules displaying the greatest Spearman's correlation with ANLN expression. MHC: major histocompatibility complex.

Figure 15(a) shows correlations between ANLN expression levels and immunoinhibitors. The immunoinhibitors displaying the greatest correlations included ADORA2A (Spearman: *ρ* = −0.511, *P* < 2.2*e* − 16), BTLA (Spearman: *ρ* = −0.342, *P* = 3.33*e* − 06), CD160 (Spearman: *ρ* = −0.457, *P* = 1.78*e* − 10), and KDR (Spearman: *ρ* = −0.329, *P* = 7.93*e* − 06) (Figure 15(b)).  Figure 15(c) shows correlations between ANLN expression and immunostimulators, and the immunostimulators displaying the greatest correlations included CD48 (Spearman: *ρ* = −0.388, *P* = 1.06*e* − 07), KLRK1 (Spearman: *ρ* = −0.39, *P* = 8.85*e* − 08), CXCL12 (Spearman: *ρ* = −0.373, *P* = 3.41*e* − 07), and NT5E (Spearman: *ρ* = 0.583, *P* = <2.2*e* − 16) (Figure 15(d)).  Figure 15(e) shows correlations between ANLN expression and MHC molecules, and the MHC molecules displaying the greatest correlations included HLA-DOA (Spearman: *ρ* = −0.259, *P* = 0.000473), HLA-DPB1 (Spearman: *ρ* = −0.355, *P* = 1.25*e* − 06), TAP1 (Spearman: *ρ* = 0.294, *P* = 6.91*e* − 05), and TAP2 (Spearman: *ρ* = 0.293, *P* = 7.3*e* − 05) (Figure 15(f)). The greatest correlations between immunomodulators and MYEOV were displayed in Figure S6. Therefore, ANLN and MYEOV may be involved regulating the above immune molecules.

## 4. Discussion

The high fatality rate of pancreatic cancer is inextricably related to its own immunosuppressive microenvironment and the obvious reduction of T cell infiltration rate in the tumor [7]. Researchers have discovered that antitumor immunotherapy may be a breakthrough in tumor therapy by targeting to enhance the host's own immunity to tumors. T cells are important components of immunotherapy against tumors. When T cells encounter specific tumor antigens, the single chain variable fragments (scFv) in the antigen recognition region will bind to tumor antigens and then directly activate T cells and stimulate the secretion of cytokines, which can attack and kill tumor cells. Chimeric antigen receptor-modified T cell (CAR-T) therapy is based on this mechanism and has been achieved good results [19]. T cells are the key effectors of the tumor immune response. Tumors that grow in an immunodeficient environment have strong immunogenicity, and solid tumors lacking T cell infiltration usually have poor prognostic significance [20]. Therefore, enhancing the recognition and killing of tumor cells by T cells is a breakthrough in immunotherapy. However, the antigens found in solid tumors so far are all tumor-associated antigens. Such antigens will also be expressed in other normal tissues, which will put a great risk in targeted therapy, called “off-target effect” [21]. Therefore, it is particularly important to choose suitable tumor-associated antigens while continuously exploring the specific antigen of pancreatic cancer. At present, the effective molecules confirmed by researchers are B7-H3, HER2, and so on [22, 23]. In this paper, machine learning is used to screen specific molecules that are highly different from ordinary tissues and to explore new molecules that are significantly related to patient clinical characteristics, prognosis, and T lymphocyte infiltration to find ideal immunotherapy targets. Our results consistently demonstrate that the five-mRNA risk signature is very robust for predicting clinical outcome of PC. And our risk signature is validated with two patient cohorts from different sources, which strongly demonstrates its robustness for predicting prognosis of PC. In addition, we have constructed a nomogram model, which is built up based on the five-mRNA risk signature.

The proprotein encoded by GZMB (granzyme B) is secreted by natural killer (NK) cells and cytotoxic T lymphocytes (CTL) [24]. CD8A encodes a glycoprotein on the surface of most CTL [25]. The GZMB/CD8A ratio can reflect the degree of immune cytotoxicity and cytotoxic T lymphocyte infiltration, which can be used to predict the response of tumor genes to immune cells [26]. Therefore, GZMB/CD8A ratio-related genes are promising new targets for immunotherapy. We have confirmed that the expressions of ANLN and MYEOV were positively correlated with GZMB/CD8A ratio, which revealed that high expressions of those genes with high T lymphocyte cell infiltration in PC. And the high expressions of ANLN and MYEOV were also related to the poorer prognoses of these patients; so, we speculated that immunotherapy may be more effective in such patients and can significantly improve the prognosis.

Type 17T helper (Th-17) cells induce immune responses by secreting IL-17, IL-21, and tumor necrosis factor-*α* (TNF-*α*) [27]. Type 2T helper (Th-2) cells can secrete cytokines such as IL-4, IL-5, IL-9, IL-10, and IL-13, stimulate the proliferation of B lymphocytes, and participate in humoral immune responses [28]. *γδ*T cells can directly recognize malignant cells and exert antitumor activity by producing various chemokines and cytokines (such as TNF-*α* and IFN-*γ*). IFN-*γ* can directly inhibit tumor growth, stimulate macrophages, and block angiogenesis [29]. CD56+ natural killer (NK) cells can express Fc receptors and mediate antibody-dependent cell-mediated cytotoxicity (ADCC) by binding to the Fc part of cancer cells Ig G, thereby inducing activation signals and killing target cells [30, 31]. Furthermore, the results from TISIDB database showed that ANLN and MYEOV had the greatest correlation with immunoinhibitors (such as ADORA2A, CD160, BTLA, and KDR), immunostimulators (such as NT5E, KLRK1, CXCL12, and CD48), and MHC molecules (such as HLA-DPB1, TAP1, HLA-DOA, and TAP2). The above results further indicated that ANLN and MYEOV are related to antitumor immune cells in the body, revealing their potential value in pancreatic cancer immunotherapy.

Our study also revealed that the CNAs of ANLN and MYEOV significantly affected the CD4+ T cell infiltration level in PC by deleting and gaining aim level, providing insight into the TIME. Therefore, we further analyzed the effects of ANLN and MYEOV level on the OS and RFS of the CD4 + T cell low infiltration group and high infiltration group, respectively. The degree of CD4 + T cell infiltration affected the RFS and OS outcomes with different ANLN expression levels and the RFS outcome with different MYEOV expression levels in PC patients, suggesting that enriched CD4+ T cell infiltration could improve patient prognosis (we have confirmed that the high expression of these two genes leading poor prognosis previously). In addition, we found that the expression levels of the two genes were significantly positively correlated. GSEA analysis revealed that the molecular pathways of ANLN and MYEOV were similar. Therefore, we speculated that these two genes may have parallel effect on the progression of pancreatic tumors.

It is necessary to point out some limitations of current research. First, the prognostic power of the five-mRNA signature was evaluated in only two external datasets. Large scale independent research is necessary to further verify the validity of this signature. Second, our own independent cohort does not provide information on other clinical features such as chemoresistance, radioresistance, and intratumoral heterogeneity. Therefore, we cannot analyze the correlation between this five-mRNA signature and the above clinical features. Further research and more detailed clinical data are needed to explore. Similarly, for ANLN and MYEOV, we need more external data and experiments to prove that they are related to the prognosis and immune microenvironment of pancreatic cancer.

## 5. Conclusions

We established a five-gene signature for the prognosis of PC by the public databases. Then, we screened ANLN and MYEOV related to prognosis and the immune microenvironment in pancreatic cancer. ANLN and MYEOV are involved in the progress of pancreatic cancer and are expected to become new markers and therapeutic targets in the future.

## Data Availability

The data used in the current study are available from the GEO (https://www.ncbi.nlm.nih.gov), TCGA (https://portal.gdc.cancer.gov/), and ICGC (https://daco.icgc.org/).

## Supplementary Materials

Supplementary Materials Supplementary figures present detailed information about the workflow of study and show that ANLN and MYEOV are related to the prognosis and immune microenvironment of pancreatic cancer. Supplementary tables give detailed information about the genes which we screened after each step.
