# Supplementary material for: Bioinformatic Analysis of Prognostic and Immune-Related Genes in Pancreatic Cancer
Source: Comput Math Methods Med. 2021 Aug 3;2021:5549298. doi: 10.1155/2021/5549298 (PMC8355962; doi:10.1155/2021/5549298)
Supplement: Supplementary Materials — Supplementary figures present detailed information about the workflow of study and show that ANLN and MYEOV are related to the prognosis and immune microenvironment of pancreatic cancer. Supplementary tables give detailed information about the genes which we screened after each step. [file 5549298.f1.docx]

SUPPLEMENTARY MATERIALS

Supplementary Figure 1. The detailed workflow of study.


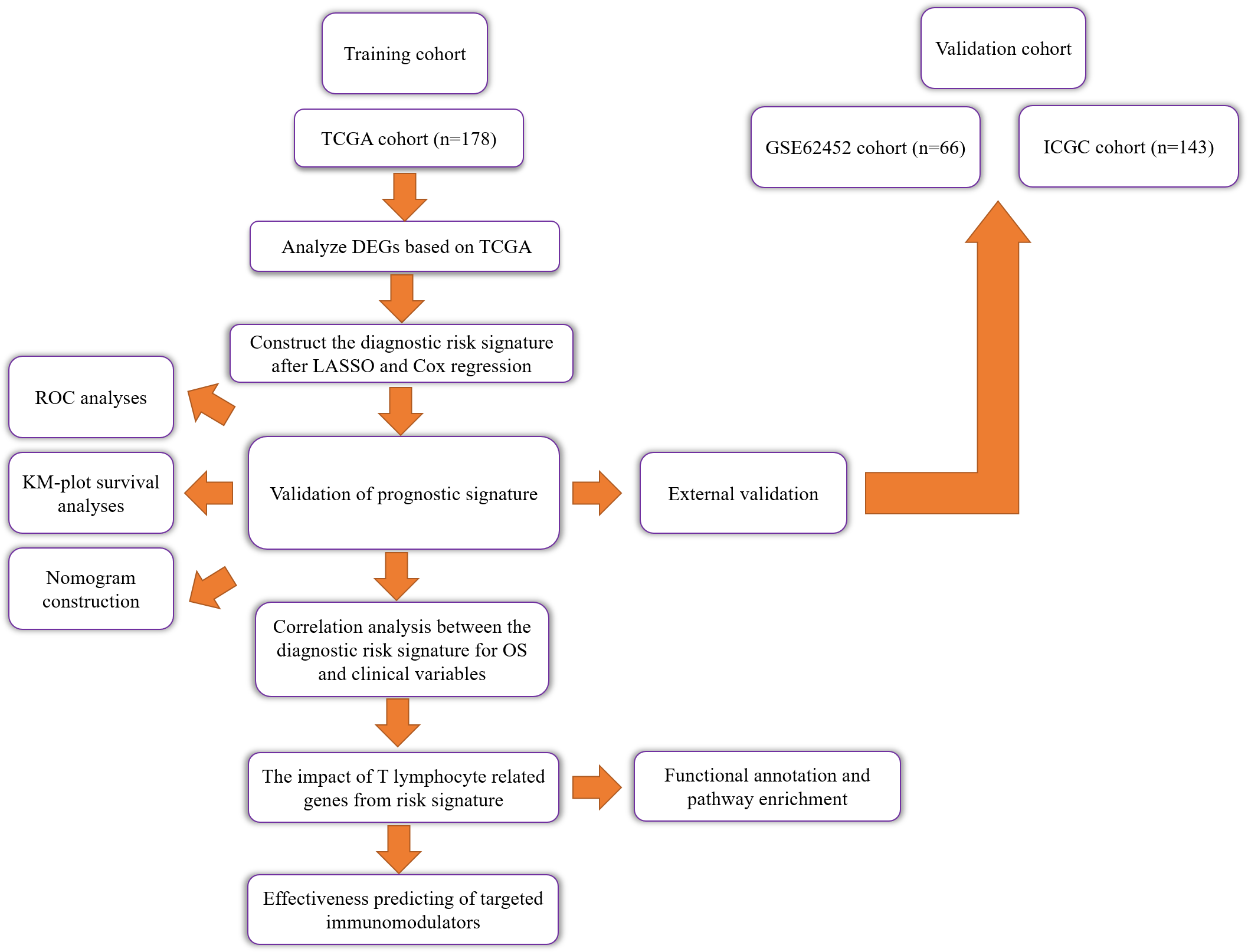


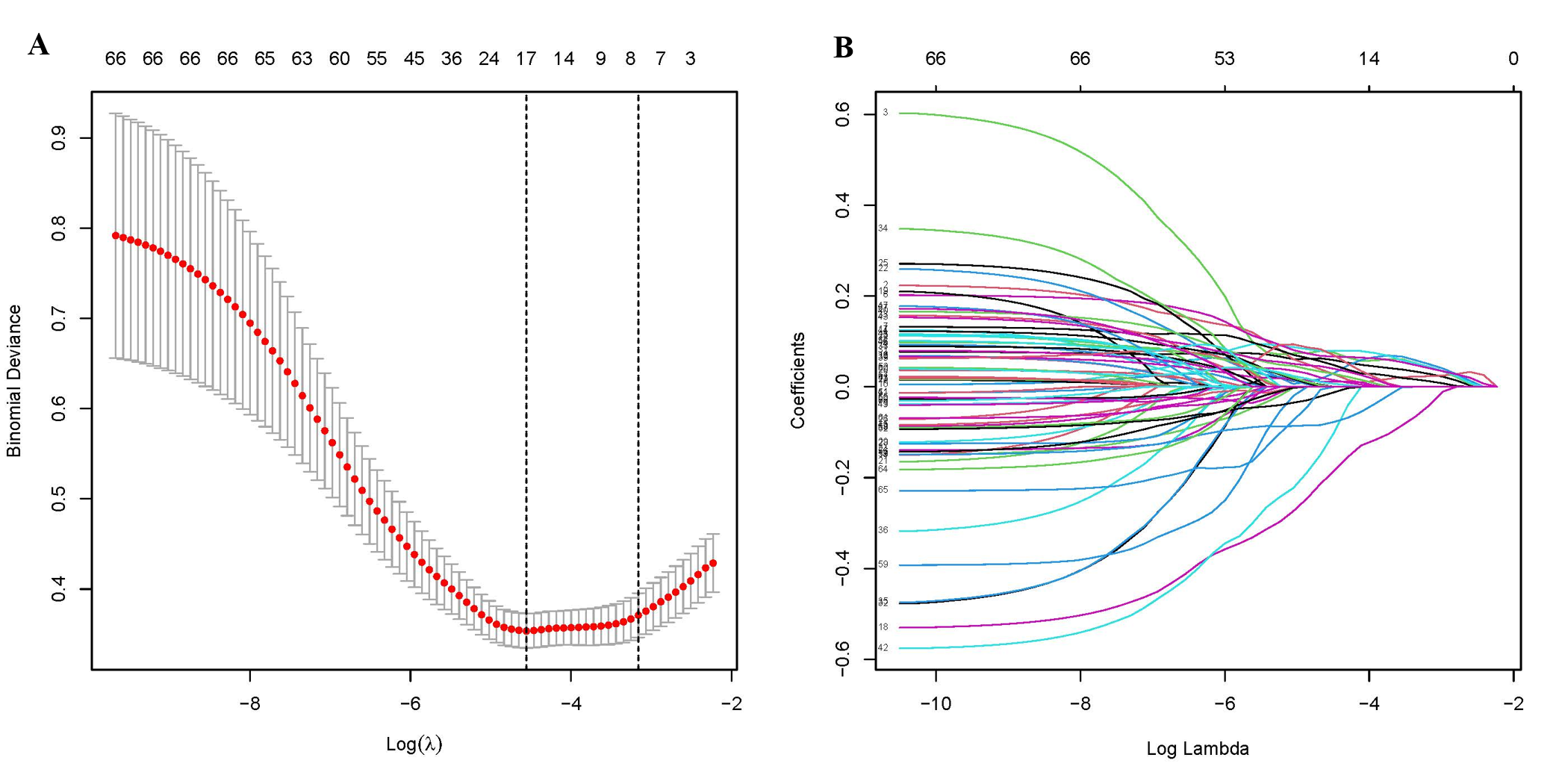


Supplementary Figure 2: Lasso regression analysis. **a** Partial likelihood deviance for tuning the parameter selection in the LASSO regression model in TCGA cohort. **b** LASSO coefficient profiles of the 67 mRNAs in TCGA cohort.


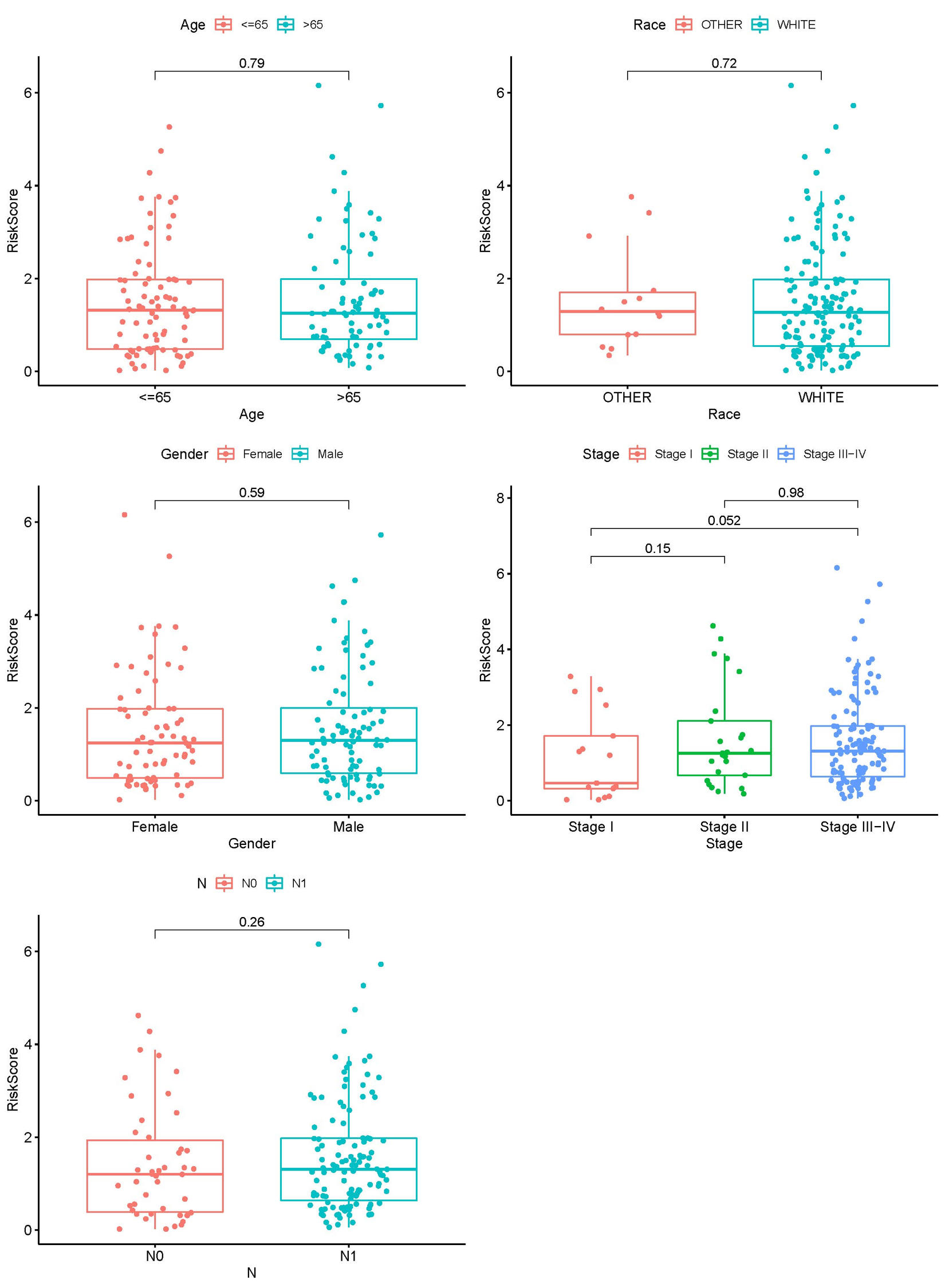


Supplementary Figure 3: Correlation of RiskScore and other clinical traits.


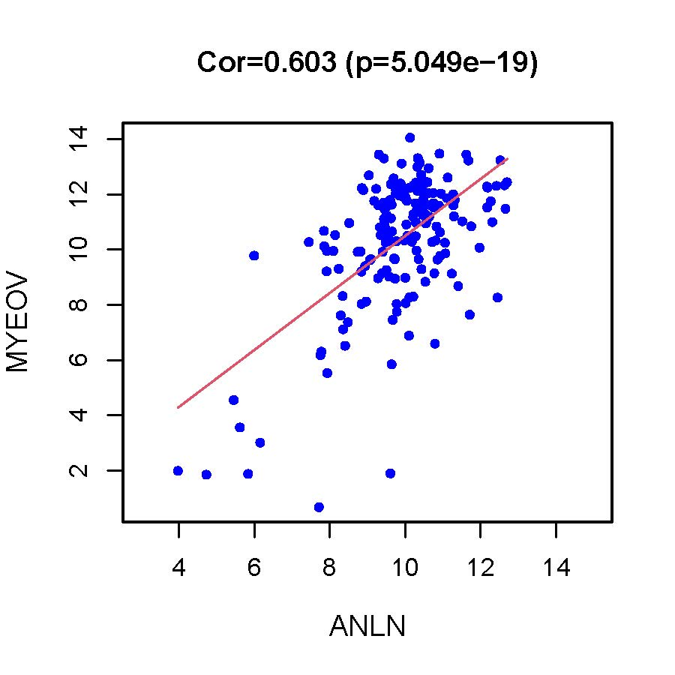


Supplementary Figure 4: Correlation analysis between ANLN and MYEOV.


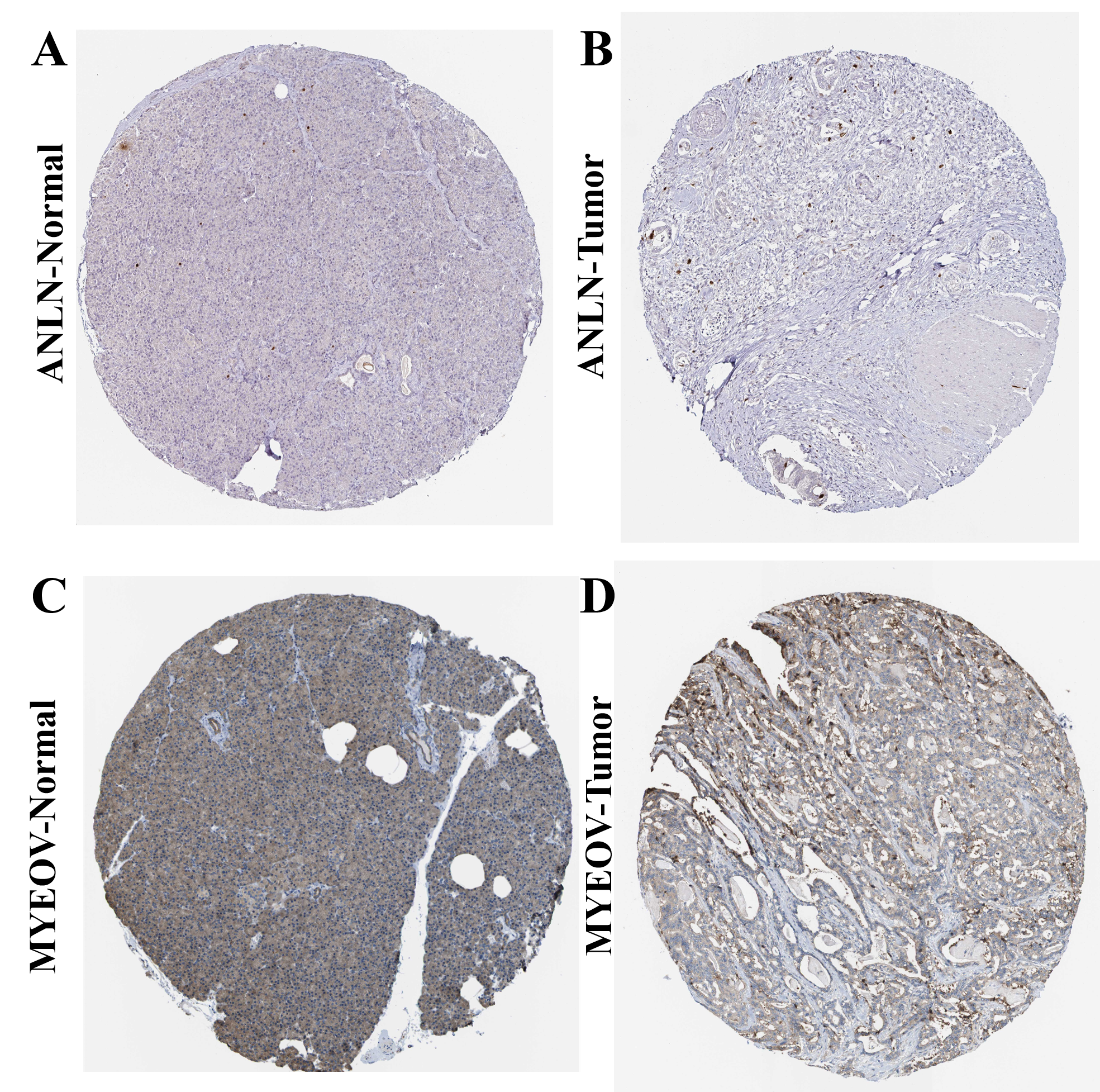


Supplementary Figure 5: Analysis of the protein expression of ANLN and MYEOV by HPA. **a-b** ANLN, **c-d** MYEOV in PC and non-cancerous pancreatic tissues derived from the HPA database. PC, pancreatic carcinoma; HPA, Human Protein Atlas.


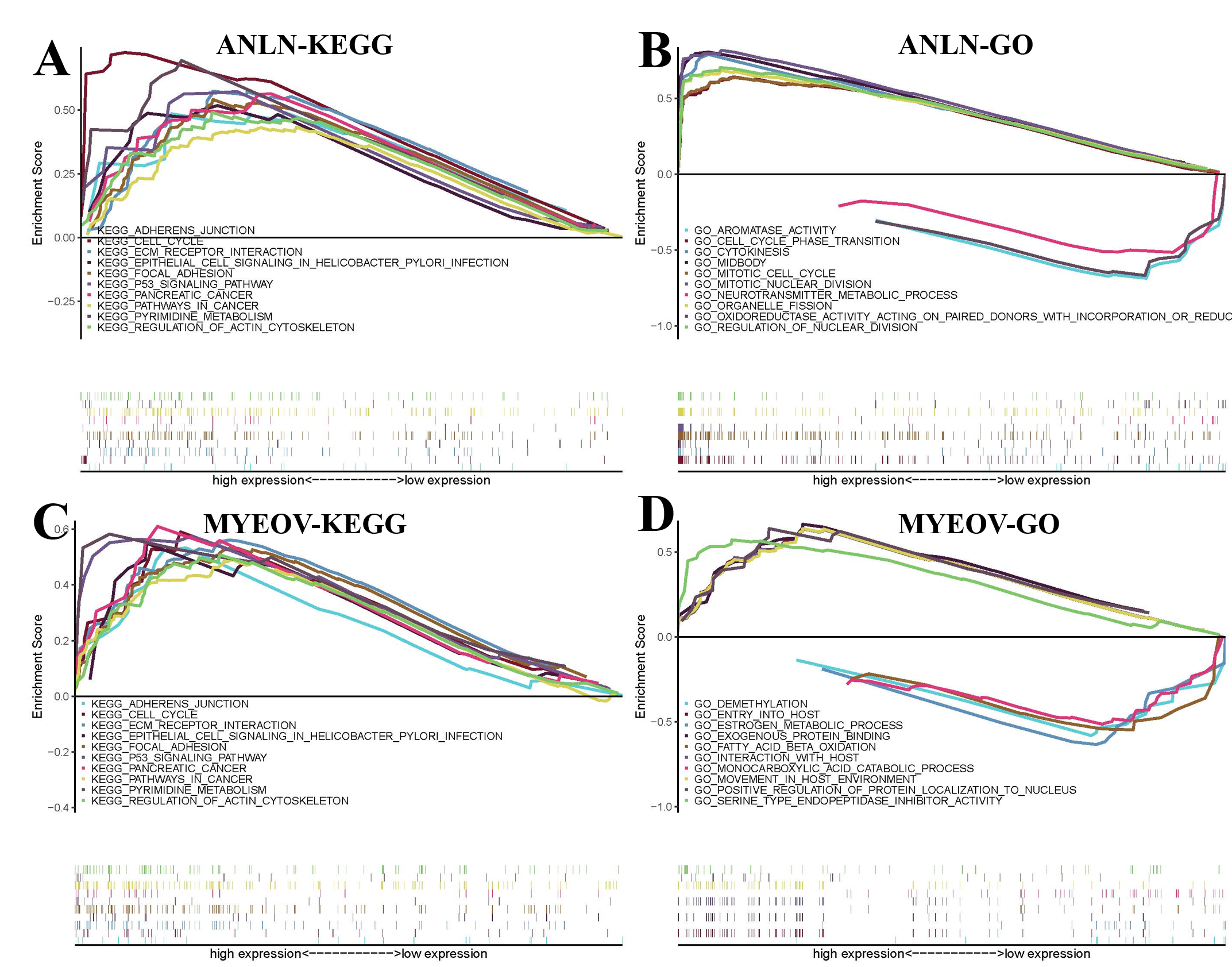


Supplementary Figure 6: Gene set enrichment analysis results based on ANLN and MYEOV. (A-B shows the results of ANLN and MYEOV, respectively).


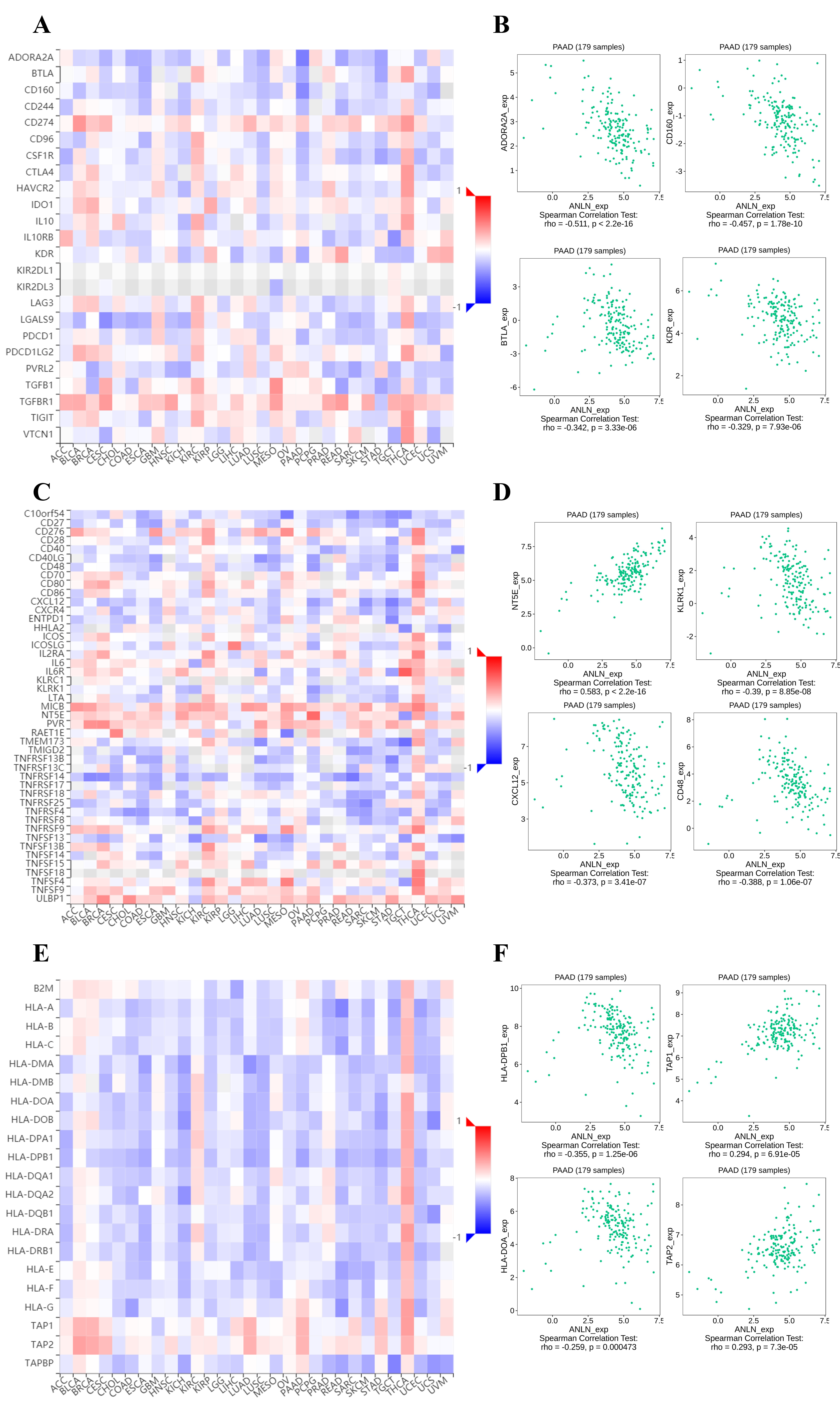


Supplementary Figure 7: Spearman’s correlation of ANLN with immunomodulators. **a** Relations between the immunoinhibitors and ANLN expression. **b** Top 4 immunoinhibitors displaying the greatest Spearman’s correlation with ANLN expression.**c** Relations between immunostimulators and ANLN expression. **d** Top 4 immunostimulators displaying the greatest Spearman’s correlation with ANLN expression. **e** Relations between MHC molecules and ANLN expression. **f** Top 4 MHC molecules displaying the greatest Spearman’s correlation with ANLN expression. MHC, major histocompatibility complex.


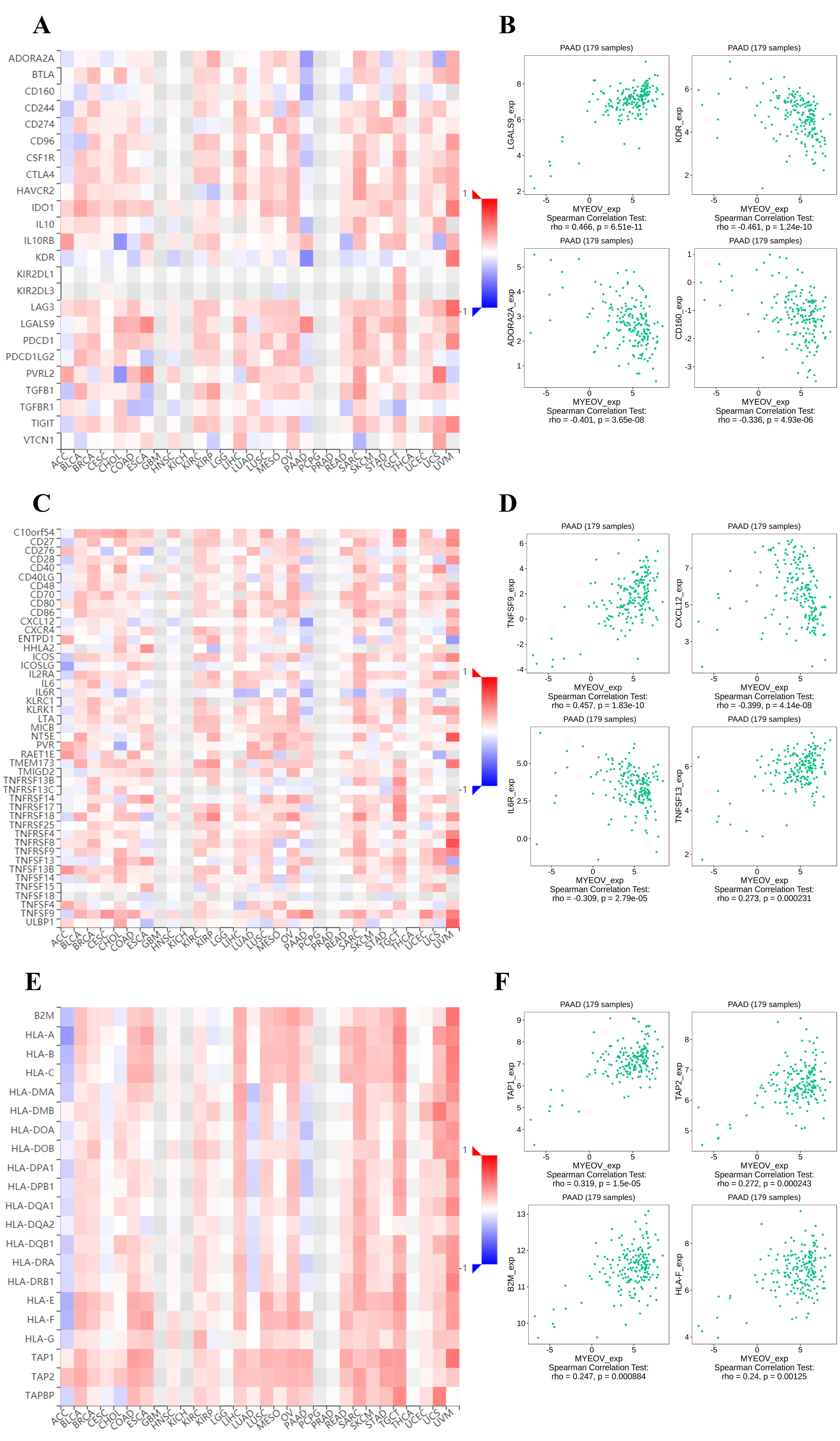


Supplementary Figure 8: Spearman’s correlation of MYEOV with immunomodulators. **a** Relations between the immunoinhibitors and MYEOV expression. **b** Top 4 immunoinhibitors displaying the greatest Spearman’s correlation with ANLN expression. **c** Relations between immunostimulators and MYEOV expression. **d** Top 4 immunostimulators displaying the greatest Spearman’s correlation with MYEOV expression. **e** Relations between MHC molecules and MYEOV expression. **f** Top 4 MHC molecules displaying the greatest Spearman’s correlation with MYEOV expression. MHC, major histocompatibility complex.

Supplementary Table S1. List of Pancancer immune metagenes.

| **Metagene** | **Cell type** | **Immunity** |
| --- | --- | --- |
| ADAM28 | Activated B cell | Adaptive |
| CD180 | Activated B cell | Adaptive |
| CD79B | Activated B cell | Adaptive |
| BLK | Activated B cell | Adaptive |
| CD19 | Activated B cell | Adaptive |
| MS4A1 | Activated B cell | Adaptive |
| TNFRSF17 | Activated B cell | Adaptive |
| IGHM | Activated B cell | Adaptive |
| GNG7 | Activated B cell | Adaptive |
| MICAL3 | Activated B cell | Adaptive |
| SPIB | Activated B cell | Adaptive |
| HLA-DOB | Activated B cell | Adaptive |
| IGKC | Activated B cell | Adaptive |
| PNOC | Activated B cell | Adaptive |
| FCRL2 | Activated B cell | Adaptive |
| BACH2 | Activated B cell | Adaptive |
| CR2 | Activated B cell | Adaptive |
| TCL1A | Activated B cell | Adaptive |
| AKNA | Activated B cell | Adaptive |
| ARHGAP25 | Activated B cell | Adaptive |
| CCL21 | Activated B cell | Adaptive |
| CD27 | Activated B cell | Adaptive |
| CD38 | Activated B cell | Adaptive |
| CLEC17A | Activated B cell | Adaptive |
| CLEC9A | Activated B cell | Adaptive |
| CLECL1 | Activated B cell | Adaptive |
| AIM2 | Activated CD4 T cell | Adaptive |
| BIRC3 | Activated CD4 T cell | Adaptive |
| BRIP1 | Activated CD4 T cell | Adaptive |
| CCL20 | Activated CD4 T cell | Adaptive |
| CCL4 | Activated CD4 T cell | Adaptive |
| CCL5 | Activated CD4 T cell | Adaptive |
| CCNB1 | Activated CD4 T cell | Adaptive |
| CCR7 | Activated CD4 T cell | Adaptive |
| DUSP2 | Activated CD4 T cell | Adaptive |
| ESCO2 | Activated CD4 T cell | Adaptive |
| ETS1 | Activated CD4 T cell | Adaptive |
| EXO1 | Activated CD4 T cell | Adaptive |
| EXOC6 | Activated CD4 T cell | Adaptive |
| IARS | Activated CD4 T cell | Adaptive |
| ITK | Activated CD4 T cell | Adaptive |
| KIF11 | Activated CD4 T cell | Adaptive |
| KNTC1 | Activated CD4 T cell | Adaptive |
| NUF2 | Activated CD4 T cell | Adaptive |
| PRC1 | Activated CD4 T cell | Adaptive |
| PSAT1 | Activated CD4 T cell | Adaptive |
| RGS1 | Activated CD4 T cell | Adaptive |
| RTKN2 | Activated CD4 T cell | Adaptive |
| SAMSN1 | Activated CD4 T cell | Adaptive |
| SELL | Activated CD4 T cell | Adaptive |
| TRAT1 | Activated CD4 T cell | Adaptive |
| ADRM1 | Activated CD8 T cell | Adaptive |
| AHSA1 | Activated CD8 T cell | Adaptive |
| C1GALT1C1 | Activated CD8 T cell | Adaptive |
| CCT6B | Activated CD8 T cell | Adaptive |
| CD37 | Activated CD8 T cell | Adaptive |
| CD3D | Activated CD8 T cell | Adaptive |
| CD3E | Activated CD8 T cell | Adaptive |
| CD3G | Activated CD8 T cell | Adaptive |
| CD69 | Activated CD8 T cell | Adaptive |
| CD8A | Activated CD8 T cell | Adaptive |
| CETN3 | Activated CD8 T cell | Adaptive |
| CSE1L | Activated CD8 T cell | Adaptive |
| GEMIN6 | Activated CD8 T cell | Adaptive |
| GNLY | Activated CD8 T cell | Adaptive |
| GPT2 | Activated CD8 T cell | Adaptive |
| GZMA | Activated CD8 T cell | Adaptive |
| GZMH | Activated CD8 T cell | Adaptive |
| GZMK | Activated CD8 T cell | Adaptive |
| IL2RB | Activated CD8 T cell | Adaptive |
| LCK | Activated CD8 T cell | Adaptive |
| MPZL1 | Activated CD8 T cell | Adaptive |
| NKG7 | Activated CD8 T cell | Adaptive |
| PIK3IP1 | Activated CD8 T cell | Adaptive |
| PTRH2 | Activated CD8 T cell | Adaptive |
| TIMM13 | Activated CD8 T cell | Adaptive |
| ZAP70 | Activated CD8 T cell | Adaptive |
| ABHD3 | Central memory CD4 T cell | Adaptive |
| AHNAK | Central memory CD4 T cell | Adaptive |
| ANXA2P2 | Central memory CD4 T cell | Adaptive |
| AQP3 | Central memory CD4 T cell | Adaptive |
| ATHL1 | Central memory CD4 T cell | Adaptive |
| BMI1 | Central memory CD4 T cell | Adaptive |
| BZW2 | Central memory CD4 T cell | Adaptive |
| CD63 | Central memory CD4 T cell | Adaptive |
| COL4A1 | Central memory CD4 T cell | Adaptive |
| CYLD | Central memory CD4 T cell | Adaptive |
| ELMO2 | Central memory CD4 T cell | Adaptive |
| FYN | Central memory CD4 T cell | Adaptive |
| GLIPR1 | Central memory CD4 T cell | Adaptive |
| GSS | Central memory CD4 T cell | Adaptive |
| IFITM2 | Central memory CD4 T cell | Adaptive |
| ITGB1 | Central memory CD4 T cell | Adaptive |
| ITGB2 | Central memory CD4 T cell | Adaptive |
| KLF5 | Central memory CD4 T cell | Adaptive |
| LSP1 | Central memory CD4 T cell | Adaptive |
| NDUFB9 | Central memory CD4 T cell | Adaptive |
| PKM2 | Central memory CD4 T cell | Adaptive |
| SFXN3 | Central memory CD4 T cell | Adaptive |
| SIRPG | Central memory CD4 T cell | Adaptive |
| SMAD4 | Central memory CD4 T cell | Adaptive |
| STX4 | Central memory CD4 T cell | Adaptive |
| TRADD | Central memory CD4 T cell | Adaptive |
| VIM | Central memory CD4 T cell | Adaptive |
| XRCC6 | Central memory CD4 T cell | Adaptive |
| ACTN4 | Central memory CD8 T cell | Adaptive |
| ADAM12 | Central memory CD8 T cell | Adaptive |
| ADCY9 | Central memory CD8 T cell | Adaptive |
| F13A1 | Central memory CD8 T cell | Adaptive |
| FCER1G | Central memory CD8 T cell | Adaptive |
| FCGR3B | Central memory CD8 T cell | Adaptive |
| FGF7 | Central memory CD8 T cell | Adaptive |
| FKBP4 | Central memory CD8 T cell | Adaptive |
| GLUD1 | Central memory CD8 T cell | Adaptive |
| GM2A | Central memory CD8 T cell | Adaptive |
| GUSB | Central memory CD8 T cell | Adaptive |
| IL1RN | Central memory CD8 T cell | Adaptive |
| NOL11 | Central memory CD8 T cell | Adaptive |
| NTRK1 | Central memory CD8 T cell | Adaptive |
| RARA | Central memory CD8 T cell | Adaptive |
| RNF128 | Central memory CD8 T cell | Adaptive |
| SIGLEC1 | Central memory CD8 T cell | Adaptive |
| TNFRSF11A | Central memory CD8 T cell | Adaptive |
| TOX4 | Central memory CD8 T cell | Adaptive |
| UBA52 | Central memory CD8 T cell | Adaptive |
| ULBP1 | Central memory CD8 T cell | Adaptive |
| ATM | Effector memeory CD4 T cell | Adaptive |
| CASP3 | Effector memeory CD4 T cell | Adaptive |
| CASQ1 | Effector memeory CD4 T cell | Adaptive |
| CD300E | Effector memeory CD4 T cell | Adaptive |
| DARS | Effector memeory CD4 T cell | Adaptive |
| DOCK9 | Effector memeory CD4 T cell | Adaptive |
| EXOSC9 | Effector memeory CD4 T cell | Adaptive |
| EZH2 | Effector memeory CD4 T cell | Adaptive |
| GDE1 | Effector memeory CD4 T cell | Adaptive |
| IL34 | Effector memeory CD4 T cell | Adaptive |
| NCOA4 | Effector memeory CD4 T cell | Adaptive |
| NEFL | Effector memeory CD4 T cell | Adaptive |
| PDGFRL | Effector memeory CD4 T cell | Adaptive |
| PTGS1 | Effector memeory CD4 T cell | Adaptive |
| REPS1 | Effector memeory CD4 T cell | Adaptive |
| SCG2 | Effector memeory CD4 T cell | Adaptive |
| SDPR | Effector memeory CD4 T cell | Adaptive |
| SIGLEC14 | Effector memeory CD4 T cell | Adaptive |
| SIGLEC6 | Effector memeory CD4 T cell | Adaptive |
| TAL1 | Effector memeory CD4 T cell | Adaptive |
| TFEC | Effector memeory CD4 T cell | Adaptive |
| TIPIN | Effector memeory CD4 T cell | Adaptive |
| TPK1 | Effector memeory CD4 T cell | Adaptive |
| UQCRB | Effector memeory CD4 T cell | Adaptive |
| USP9Y | Effector memeory CD4 T cell | Adaptive |
| WIPF1 | Effector memeory CD4 T cell | Adaptive |
| ZCRB1 | Effector memeory CD4 T cell | Adaptive |
| ACAP1 | Effector memeory CD8 T cell | Adaptive |
| APOL3 | Effector memeory CD8 T cell | Adaptive |
| ARHGAP10 | Effector memeory CD8 T cell | Adaptive |
| ATP10D | Effector memeory CD8 T cell | Adaptive |
| C3AR1 | Effector memeory CD8 T cell | Adaptive |
| CCR5 | Effector memeory CD8 T cell | Adaptive |
| CD160 | Effector memeory CD8 T cell | Adaptive |
| CD55 | Effector memeory CD8 T cell | Adaptive |
| CFLAR | Effector memeory CD8 T cell | Adaptive |
| CMKLR1 | Effector memeory CD8 T cell | Adaptive |
| DAPP1 | Effector memeory CD8 T cell | Adaptive |
| FCRL6 | Effector memeory CD8 T cell | Adaptive |
| FLT3LG | Effector memeory CD8 T cell | Adaptive |
| GZMM | Effector memeory CD8 T cell | Adaptive |
| HAPLN3 | Effector memeory CD8 T cell | Adaptive |
| HLA-DMB | Effector memeory CD8 T cell | Adaptive |
| HLA-DPA1 | Effector memeory CD8 T cell | Adaptive |
| HLA-DPB1 | Effector memeory CD8 T cell | Adaptive |
| IFI16 | Effector memeory CD8 T cell | Adaptive |
| LIME1 | Effector memeory CD8 T cell | Adaptive |
| LTK | Effector memeory CD8 T cell | Adaptive |
| NFKBIA | Effector memeory CD8 T cell | Adaptive |
| SETD7 | Effector memeory CD8 T cell | Adaptive |
| SIK1 | Effector memeory CD8 T cell | Adaptive |
| TRIB2 | Effector memeory CD8 T cell | Adaptive |
| ACP5 | Gamma delta T cell | Adaptive |
| AQP9 | Gamma delta T cell | Adaptive |
| BTN3A2 | Gamma delta T cell | Adaptive |
| C1orf54 | Gamma delta T cell | Adaptive |
| CARD8 | Gamma delta T cell | Adaptive |
| CCL18 | Gamma delta T cell | Adaptive |
| CD209 | Gamma delta T cell | Adaptive |
| CD33 | Gamma delta T cell | Adaptive |
| CD36 | Gamma delta T cell | Adaptive |
| CDK5 | Gamma delta T cell | Adaptive |
| IL10RB | Gamma delta T cell | Adaptive |
| KLRF1 | Gamma delta T cell | Adaptive |
| LGALS1 | Gamma delta T cell | Adaptive |
| MAPK7 | Gamma delta T cell | Adaptive |
| KLHL7 | Gamma delta T cell | Adaptive |
| KRT80 | Gamma delta T cell | Adaptive |
| LAMC1 | Gamma delta T cell | Adaptive |
| LCORL | Gamma delta T cell | Adaptive |
| LMNB1 | Gamma delta T cell | Adaptive |
| MEIS3P1 | Gamma delta T cell | Adaptive |
| MPL | Gamma delta T cell | Adaptive |
| FABP1 | Gamma delta T cell | Adaptive |
| FABP5 | Gamma delta T cell | Adaptive |
| FADD | Gamma delta T cell | Adaptive |
| MFAP3L | Gamma delta T cell | Adaptive |
| MINPP1 | Gamma delta T cell | Adaptive |
| RPS24 | Gamma delta T cell | Adaptive |
| RPS7 | Gamma delta T cell | Adaptive |
| RPS9 | Gamma delta T cell | Adaptive |
| DBNL | Gamma delta T cell | Adaptive |
| CCL13 | Gamma delta T cell | Adaptive |
| CD22 | Immature B cell | Adaptive |
| CYBB | Immature B cell | Adaptive |
| FAM129C | Immature B cell | Adaptive |
| FCRL1 | Immature B cell | Adaptive |
| FCRL3 | Immature B cell | Adaptive |
| FCRL5 | Immature B cell | Adaptive |
| FCRLA | Immature B cell | Adaptive |
| HDAC9 | Immature B cell | Adaptive |
| HLA-DQA1 | Immature B cell | Adaptive |
| HVCN1 | Immature B cell | Adaptive |
| KIAA0226 | Immature B cell | Adaptive |
| NCF1 | Immature B cell | Adaptive |
| NCF1B | Immature B cell | Adaptive |
| P2RY10 | Immature B cell | Adaptive |
| SP100 | Immature B cell | Adaptive |
| TXNIP | Immature B cell | Adaptive |
| STAP1 | Immature B cell | Adaptive |
| TAGAP | Immature B cell | Adaptive |
| ZCCHC2 | Immature B cell | Adaptive |
| AICDA | Memory B cell | Adaptive |
| CCNA2 | Memory B cell | Adaptive |
| CDKN3 | Memory B cell | Adaptive |
| CLCN5 | Memory B cell | Adaptive |
| ENPP1 | Memory B cell | Adaptive |
| FCER1A | Memory B cell | Adaptive |
| FCRL4 | Memory B cell | Adaptive |
| MYC | Memory B cell | Adaptive |
| RUNX2 | Memory B cell | Adaptive |
| SORL1 | Memory B cell | Adaptive |
| SOX5 | Memory B cell | Adaptive |
| STAT5A | Memory B cell | Adaptive |
| STAT5B | Memory B cell | Adaptive |
| TLR9 | Memory B cell | Adaptive |
| CCL3L1 | Regulatory T cell | Adaptive |
| CD72 | Regulatory T cell | Adaptive |
| CLEC5A | Regulatory T cell | Adaptive |
| FOXP3 | Regulatory T cell | Adaptive |
| ITGA4 | Regulatory T cell | Adaptive |
| L1CAM | Regulatory T cell | Adaptive |
| LIPA | Regulatory T cell | Adaptive |
| LRP1 | Regulatory T cell | Adaptive |
| LRRC42 | Regulatory T cell | Adaptive |
| MARCO | Regulatory T cell | Adaptive |
| MMP12 | Regulatory T cell | Adaptive |
| MNDA | Regulatory T cell | Adaptive |
| MRC1 | Regulatory T cell | Adaptive |
| MS4A6A | Regulatory T cell | Adaptive |
| PELO | Regulatory T cell | Adaptive |
| PLEK | Regulatory T cell | Adaptive |
| PRSS23 | Regulatory T cell | Adaptive |
| PTGIR | Regulatory T cell | Adaptive |
| ST8SIA4 | Regulatory T cell | Adaptive |
| STAB1 | Regulatory T cell | Adaptive |
| B3GAT1 | T follicular helper cell | Adaptive |
| CDK5R1 | T follicular helper cell | Adaptive |
| PDCD1 | T follicular helper cell | Adaptive |
| BCL6 | T follicular helper cell | Adaptive |
| CD200 | T follicular helper cell | Adaptive |
| CD83 | T follicular helper cell | Adaptive |
| CD84 | T follicular helper cell | Adaptive |
| FGF2 | T follicular helper cell | Adaptive |
| GPR18 | T follicular helper cell | Adaptive |
| CEBPA | T follicular helper cell | Adaptive |
| CECR1 | T follicular helper cell | Adaptive |
| CLEC10A | T follicular helper cell | Adaptive |
| CLEC4A | T follicular helper cell | Adaptive |
| CSF1R | T follicular helper cell | Adaptive |
| CTSS | T follicular helper cell | Adaptive |
| DMN | T follicular helper cell | Adaptive |
| DPP4 | T follicular helper cell | Adaptive |
| LRRC32 | T follicular helper cell | Adaptive |
| MC5R | T follicular helper cell | Adaptive |
| MICA | T follicular helper cell | Adaptive |
| NCAM1 | T follicular helper cell | Adaptive |
| NCR2 | T follicular helper cell | Adaptive |
| NRP1 | T follicular helper cell | Adaptive |
| PDCD1LG2 | T follicular helper cell | Adaptive |
| PDCD6 | T follicular helper cell | Adaptive |
| PRDX1 | T follicular helper cell | Adaptive |
| RAE1 | T follicular helper cell | Adaptive |
| RAET1E | T follicular helper cell | Adaptive |
| SIGLEC7 | T follicular helper cell | Adaptive |
| SIGLEC9 | T follicular helper cell | Adaptive |
| TYRO3 | T follicular helper cell | Adaptive |
| CHST12 | T follicular helper cell | Adaptive |
| CLIC3 | T follicular helper cell | Adaptive |
| IVNS1ABP | T follicular helper cell | Adaptive |
| KIR2DL2 | T follicular helper cell | Adaptive |
| LGMN | T follicular helper cell | Adaptive |
| CD70 | Type 1 T helper cell | Adaptive |
| TBX21 | Type 1 T helper cell | Adaptive |
| ADAM8 | Type 1 T helper cell | Adaptive |
| AHCYL2 | Type 1 T helper cell | Adaptive |
| ALCAM | Type 1 T helper cell | Adaptive |
| B3GALNT1 | Type 1 T helper cell | Adaptive |
| BBS12 | Type 1 T helper cell | Adaptive |
| BST1 | Type 1 T helper cell | Adaptive |
| CD151 | Type 1 T helper cell | Adaptive |
| CD47 | Type 1 T helper cell | Adaptive |
| CD48 | Type 1 T helper cell | Adaptive |
| CD52 | Type 1 T helper cell | Adaptive |
| CD53 | Type 1 T helper cell | Adaptive |
| CD59 | Type 1 T helper cell | Adaptive |
| CD6 | Type 1 T helper cell | Adaptive |
| CD68 | Type 1 T helper cell | Adaptive |
| CD7 | Type 1 T helper cell | Adaptive |
| CD96 | Type 1 T helper cell | Adaptive |
| CFHR3 | Type 1 T helper cell | Adaptive |
| CHRM3 | Type 1 T helper cell | Adaptive |
| CLEC7A | Type 1 T helper cell | Adaptive |
| COL23A1 | Type 1 T helper cell | Adaptive |
| COL4A4 | Type 1 T helper cell | Adaptive |
| COL5A3 | Type 1 T helper cell | Adaptive |
| DAB1 | Type 1 T helper cell | Adaptive |
| DLEU7 | Type 1 T helper cell | Adaptive |
| DOC2B | Type 1 T helper cell | Adaptive |
| EMP1 | Type 1 T helper cell | Adaptive |
| F12 | Type 1 T helper cell | Adaptive |
| FURIN | Type 1 T helper cell | Adaptive |
| GAB3 | Type 1 T helper cell | Adaptive |
| GATM | Type 1 T helper cell | Adaptive |
| GFPT2 | Type 1 T helper cell | Adaptive |
| GPR25 | Type 1 T helper cell | Adaptive |
| GREM2 | Type 1 T helper cell | Adaptive |
| HAVCR1 | Type 1 T helper cell | Adaptive |
| HSD11B1 | Type 1 T helper cell | Adaptive |
| HUNK | Type 1 T helper cell | Adaptive |
| IGF2 | Type 1 T helper cell | Adaptive |
| RCSD1 | Type 1 T helper cell | Adaptive |
| RYR1 | Type 1 T helper cell | Adaptive |
| SAV1 | Type 1 T helper cell | Adaptive |
| SELE | Type 1 T helper cell | Adaptive |
| SELP | Type 1 T helper cell | Adaptive |
| SH3KBP1 | Type 1 T helper cell | Adaptive |
| SIT1 | Type 1 T helper cell | Adaptive |
| SLC35B3 | Type 1 T helper cell | Adaptive |
| SIGLEC10 | Type 1 T helper cell | Adaptive |
| SKAP1 | Type 1 T helper cell | Adaptive |
| THUMPD2 | Type 1 T helper cell | Adaptive |
| TIGIT | Type 1 T helper cell | Adaptive |
| ZEB2 | Type 1 T helper cell | Adaptive |
| ENC1 | Type 1 T helper cell | Adaptive |
| FAM134B | Type 1 T helper cell | Adaptive |
| FBXO30 | Type 1 T helper cell | Adaptive |
| FCGR2C | Type 1 T helper cell | Adaptive |
| STAC | Type 1 T helper cell | Adaptive |
| LTC4S | Type 1 T helper cell | Adaptive |
| MAN1B1 | Type 1 T helper cell | Adaptive |
| MDH1 | Type 1 T helper cell | Adaptive |
| MMD | Type 1 T helper cell | Adaptive |
| RGS16 | Type 1 T helper cell | Adaptive |
| IL12A | Type 1 T helper cell | Adaptive |
| P2RX5 | Type 1 T helper cell | Adaptive |
| CD97 | Type 1 T helper cell | Adaptive |
| ITGB4 | Type 1 T helper cell | Adaptive |
| ICAM3 | Type 1 T helper cell | Adaptive |
| METRNL | Type 1 T helper cell | Adaptive |
| TNFRSF1A | Type 1 T helper cell | Adaptive |
| IRF1 | Type 1 T helper cell | Adaptive |
| HTR2B | Type 1 T helper cell | Adaptive |
| CALD1 | Type 1 T helper cell | Adaptive |
| MOCOS | Type 1 T helper cell | Adaptive |
| TRAF3IP2 | Type 1 T helper cell | Adaptive |
| TLR8 | Type 1 T helper cell | Adaptive |
| TRAF1 | Type 1 T helper cell | Adaptive |
| DUSP14 | Type 1 T helper cell | Adaptive |
| IL17A | Type 17 T helper cell | Adaptive |
| IL17RA | Type 17 T helper cell | Adaptive |
| C2CD4A | Type 17 T helper cell | Adaptive |
| C2CD4B | Type 17 T helper cell | Adaptive |
| CA2 | Type 17 T helper cell | Adaptive |
| CCDC65 | Type 17 T helper cell | Adaptive |
| CEACAM3 | Type 17 T helper cell | Adaptive |
| IL17C | Type 17 T helper cell | Adaptive |
| IL17F | Type 17 T helper cell | Adaptive |
| IL17RC | Type 17 T helper cell | Adaptive |
| IL17RE | Type 17 T helper cell | Adaptive |
| IL23A | Type 17 T helper cell | Adaptive |
| ILDR1 | Type 17 T helper cell | Adaptive |
| LONRF3 | Type 17 T helper cell | Adaptive |
| SH2D6 | Type 17 T helper cell | Adaptive |
| TNIP2 | Type 17 T helper cell | Adaptive |
| ABCA1 | Type 17 T helper cell | Adaptive |
| ABCB1 | Type 17 T helper cell | Adaptive |
| ADAMTS12 | Type 17 T helper cell | Adaptive |
| ANK1 | Type 17 T helper cell | Adaptive |
| ANKRD22 | Type 17 T helper cell | Adaptive |
| B3GALT2 | Type 17 T helper cell | Adaptive |
| CAMTA1 | Type 17 T helper cell | Adaptive |
| CCR9 | Type 17 T helper cell | Adaptive |
| CD40 | Type 17 T helper cell | Adaptive |
| GPR44 | Type 17 T helper cell | Adaptive |
| IFT80 | Type 17 T helper cell | Adaptive |
| ASB2 | Type 2 T helper cell | Adaptive |
| CSRP2 | Type 2 T helper cell | Adaptive |
| DAPK1 | Type 2 T helper cell | Adaptive |
| DLC1 | Type 2 T helper cell | Adaptive |
| DNAJC12 | Type 2 T helper cell | Adaptive |
| DUSP6 | Type 2 T helper cell | Adaptive |
| GNAI1 | Type 2 T helper cell | Adaptive |
| LAMP3 | Type 2 T helper cell | Adaptive |
| NRP2 | Type 2 T helper cell | Adaptive |
| OSBPL1A | Type 2 T helper cell | Adaptive |
| PDE4B | Type 2 T helper cell | Adaptive |
| PHLDA1 | Type 2 T helper cell | Adaptive |
| PLA2G4A | Type 2 T helper cell | Adaptive |
| RAB27B | Type 2 T helper cell | Adaptive |
| RBMS3 | Type 2 T helper cell | Adaptive |
| RNF125 | Type 2 T helper cell | Adaptive |
| TMPRSS3 | Type 2 T helper cell | Adaptive |
| GATA3 | Type 2 T helper cell | Adaptive |
| BIRC5 | Type 2 T helper cell | Adaptive |
| CDC25C | Type 2 T helper cell | Adaptive |
| CDC7 | Type 2 T helper cell | Adaptive |
| CENPF | Type 2 T helper cell | Adaptive |
| CXCR6 | Type 2 T helper cell | Adaptive |
| DHFR | Type 2 T helper cell | Adaptive |
| EVI5 | Type 2 T helper cell | Adaptive |
| GSTA4 | Type 2 T helper cell | Adaptive |
| HELLS | Type 2 T helper cell | Adaptive |
| IL26 | Type 2 T helper cell | Adaptive |
| LAIR2 | Type 2 T helper cell | Adaptive |
| ABCD1 | Activated dendritic cell | Innate |
| C1QC | Activated dendritic cell | Innate |
| CAPG | Activated dendritic cell | Innate |
| CCL3L3 | Activated dendritic cell | Innate |
| CD207 | Activated dendritic cell | Innate |
| CD302 | Activated dendritic cell | Innate |
| ATP5B | Activated dendritic cell | Innate |
| ATP5L | Activated dendritic cell | Innate |
| ATP6V1A | Activated dendritic cell | Innate |
| BCL2L1 | Activated dendritic cell | Innate |
| C1QB | Activated dendritic cell | Innate |
| SNURF | Activated dendritic cell | Innate |
| SPCS3 | Activated dendritic cell | Innate |
| CCNA1 | Activated dendritic cell | Innate |
| CEACAM8 | Activated dendritic cell | Innate |
| NOS2 | Activated dendritic cell | Innate |
| SRA1 | Activated dendritic cell | Innate |
| TNFRSF6B | Activated dendritic cell | Innate |
| TREM1 | Activated dendritic cell | Innate |
| TREML1 | Activated dendritic cell | Innate |
| RHOA | Activated dendritic cell | Innate |
| SLC25A37 | Activated dendritic cell | Innate |
| TNFSF14 | Activated dendritic cell | Innate |
| TREML4 | Activated dendritic cell | Innate |
| VNN2 | Activated dendritic cell | Innate |
| XPO6 | Activated dendritic cell | Innate |
| CLEC4C | Activated dendritic cell | Innate |
| TNFAIP2 | Activated dendritic cell | Innate |
| UBD | Activated dendritic cell | Innate |
| ACTR3 | Activated dendritic cell | Innate |
| RAB1A | Activated dendritic cell | Innate |
| SLA | Activated dendritic cell | Innate |
| HLA-DQA2 | Activated dendritic cell | Innate |
| SIGLEC5 | Activated dendritic cell | Innate |
| SLAMF9 | Activated dendritic cell | Innate |
| ABAT | CD56bright natural killer cell | Innate |
| C11orf75 | CD56bright natural killer cell | Innate |
| C5orf15 | CD56bright natural killer cell | Innate |
| CDHR1 | CD56bright natural killer cell | Innate |
| DCAF12 | CD56bright natural killer cell | Innate |
| DYNLL1 | CD56bright natural killer cell | Innate |
| GPR137B | CD56bright natural killer cell | Innate |
| HCP5 | CD56bright natural killer cell | Innate |
| HDGFRP2 | CD56bright natural killer cell | Innate |
| KRT86 | CD56bright natural killer cell | Innate |
| MLST8 | CD56bright natural killer cell | Innate |
| ELMOD3 | CD56bright natural killer cell | Innate |
| ENTPD5 | CD56bright natural killer cell | Innate |
| FAM119A | CD56bright natural killer cell | Innate |
| FAM179A | CD56bright natural killer cell | Innate |
| CLIC2 | CD56bright natural killer cell | Innate |
| COX7A2L | CD56bright natural killer cell | Innate |
| CREB3L4 | CD56bright natural killer cell | Innate |
| CSF1 | CD56bright natural killer cell | Innate |
| CSNK2A2 | CD56bright natural killer cell | Innate |
| CSTA | CD56bright natural killer cell | Innate |
| CSTB | CD56bright natural killer cell | Innate |
| CTPS | CD56bright natural killer cell | Innate |
| CTSD | CD56bright natural killer cell | Innate |
| FST | CD56bright natural killer cell | Innate |
| GATA2 | CD56bright natural killer cell | Innate |
| GMPR | CD56bright natural killer cell | Innate |
| HDC | CD56bright natural killer cell | Innate |
| HEY1 | CD56bright natural killer cell | Innate |
| HOXA1 | CD56bright natural killer cell | Innate |
| HS2ST1 | CD56bright natural killer cell | Innate |
| HS3ST1 | CD56bright natural killer cell | Innate |
| BCL11B | CD56bright natural killer cell | Innate |
| CDH3 | CD56bright natural killer cell | Innate |
| MYL6B | CD56bright natural killer cell | Innate |
| NAA16 | CD56bright natural killer cell | Innate |
| ClQA | CD56bright natural killer cell | Innate |
| ClQB | CD56bright natural killer cell | Innate |
| CYP27B1 | CD56bright natural killer cell | Innate |
| EIF3M | CD56bright natural killer cell | Innate |
| CYP27A1 | CD56dim natural killer cell | Innate |
| DDX55 | CD56dim natural killer cell | Innate |
| DYRK2 | CD56dim natural killer cell | Innate |
| RPL37A | CD56dim natural killer cell | Innate |
| NOTCH3 | CD56dim natural killer cell | Innate |
| AKR7A3 | CD56dim natural killer cell | Innate |
| GPRC5C | CD56dim natural killer cell | Innate |
| GRIN1 | CD56dim natural killer cell | Innate |
| HLA-E | CD56dim natural killer cell | Innate |
| PORCN | CD56dim natural killer cell | Innate |
| PSMC4 | CD56dim natural killer cell | Innate |
| UPP1 | CD56dim natural killer cell | Innate |
| IL21R | CD56dim natural killer cell | Innate |
| KIR2DS1 | CD56dim natural killer cell | Innate |
| KIR2DS2 | CD56dim natural killer cell | Innate |
| KIR2DS5 | CD56dim natural killer cell | Innate |
| GIPR | Eosinophil | Innate |
| KRT18P50 | Eosinophil | Innate |
| LRMP | Eosinophil | Innate |
| FOSB | Eosinophil | Innate |
| RRP12 | Eosinophil | Innate |
| GPR183 | Eosinophil | Innate |
| NR4A3 | Eosinophil | Innate |
| ST3GAL6 | Eosinophil | Innate |
| DEPDC5 | Eosinophil | Innate |
| PDE6C | Eosinophil | Innate |
| PKD2L2 | Eosinophil | Innate |
| GPR65 | Eosinophil | Innate |
| IL5RA | Eosinophil | Innate |
| P2RY14 | Eosinophil | Innate |
| DACH1 | Eosinophil | Innate |
| DAPK2 | Eosinophil | Innate |
| EMR3 | Eosinophil | Innate |
| ACADM | Immature dendritic cell | Innate |
| AHCYL1 | Immature dendritic cell | Innate |
| ALDH1A2 | Immature dendritic cell | Innate |
| ALDH3A2 | Immature dendritic cell | Innate |
| ALDH9A1 | Immature dendritic cell | Innate |
| ALOX15 | Immature dendritic cell | Innate |
| AMT | Immature dendritic cell | Innate |
| ARL1 | Immature dendritic cell | Innate |
| ATIC | Immature dendritic cell | Innate |
| ATP5A1 | Immature dendritic cell | Innate |
| CAPZA1 | Immature dendritic cell | Innate |
| LILRA5 | Immature dendritic cell | Innate |
| RDX | Immature dendritic cell | Innate |
| RRAGD | Immature dendritic cell | Innate |
| TACSTD2 | Immature dendritic cell | Innate |
| INPP5F | Immature dendritic cell | Innate |
| RAB38 | Immature dendritic cell | Innate |
| PLAU | Immature dendritic cell | Innate |
| CSF3R | Immature dendritic cell | Innate |
| SLC18A2 | Immature dendritic cell | Innate |
| AMPD2 | Immature dendritic cell | Innate |
| CLTB | Immature dendritic cell | Innate |
| C1orf162 | Immature dendritic cell | Innate |
| AIF1 | Macrophage | Innate |
| CCL1 | Macrophage | Innate |
| CCL14 | Macrophage | Innate |
| CCL23 | Macrophage | Innate |
| CCL26 | Macrophage | Innate |
| CD300LB | Macrophage | Innate |
| CNR1 | Macrophage | Innate |
| CNR2 | Macrophage | Innate |
| EIF1 | Macrophage | Innate |
| EIF4A1 | Macrophage | Innate |
| FPR1 | Macrophage | Innate |
| FPR2 | Macrophage | Innate |
| FRAT2 | Macrophage | Innate |
| GPR27 | Macrophage | Innate |
| GPR77 | Macrophage | Innate |
| RNASE2 | Macrophage | Innate |
| MS4A2 | Macrophage | Innate |
| BASP1 | Macrophage | Innate |
| IGSF6 | Macrophage | Innate |
| HK3 | Macrophage | Innate |
| VNN1 | Macrophage | Innate |
| FES | Macrophage | Innate |
| NPL | Macrophage | Innate |
| FZD2 | Macrophage | Innate |
| FAM198B | Macrophage | Innate |
| HNMT | Macrophage | Innate |
| SLC15A3 | Macrophage | Innate |
| CD4 | Macrophage | Innate |
| TXNDC3 | Macrophage | Innate |
| FRMD4A | Macrophage | Innate |
| CRYBB1 | Macrophage | Innate |
| HRH1 | Macrophage | Innate |
| WNT5B | Macrophage | Innate |
| ADAMTS3 | Mast cell | Innate |
| CPA3 | Mast cell | Innate |
| CMA1 | Mast cell | Innate |
| CTSG | Mast cell | Innate |
| ARHGAP15 | Mast cell | Innate |
| CPM | Mast cell | Innate |
| FCN1 | Mast cell | Innate |
| FTL | Mast cell | Innate |
| HSPA6 | Mast cell | Innate |
| ITGA9 | Mast cell | Innate |
| RNASE3 | Mast cell | Innate |
| S100A4 | Mast cell | Innate |
| SIGLEC8 | Mast cell | Innate |
| SLC6A4 | Mast cell | Innate |
| PTGS2 | Mast cell | Innate |
| EGR3 | Mast cell | Innate |
| PILRA | Mast cell | Innate |
| CCR2 | MDSC | Innate |
| CD14 | MDSC | Innate |
| CD2 | MDSC | Innate |
| CD86 | MDSC | Innate |
| CXCR4 | MDSC | Innate |
| FCGR2A | MDSC | Innate |
| FCGR2B | MDSC | Innate |
| FCGR3A | MDSC | Innate |
| FERMT3 | MDSC | Innate |
| GPSM3 | MDSC | Innate |
| IL18BP | MDSC | Innate |
| IL4R | MDSC | Innate |
| ITGAL | MDSC | Innate |
| ITGAM | MDSC | Innate |
| PARVG | MDSC | Innate |
| PSAP | MDSC | Innate |
| PTGER2 | MDSC | Innate |
| PTGES2 | MDSC | Innate |
| S100A8 | MDSC | Innate |
| S100A9 | MDSC | Innate |
| ASGR2 | Monocyte | Innate |
| CFP | Monocyte | Innate |
| ASGR1 | Monocyte | Innate |
| CD1D | Monocyte | Innate |
| UPK3A | Monocyte | Innate |
| ACTG1 | Monocyte | Innate |
| ANXA5 | Monocyte | Innate |
| ATP6V1B2 | Monocyte | Innate |
| CFL1 | Monocyte | Innate |
| DAZAP2 | Monocyte | Innate |
| CTBS | Monocyte | Innate |
| EMR4P | Monocyte | Innate |
| HIVEP2 | Monocyte | Innate |
| MARCKSL1 | Monocyte | Innate |
| MBP | Monocyte | Innate |
| MMP15 | Monocyte | Innate |
| PNPLA6 | Monocyte | Innate |
| TMBIM6 | Monocyte | Innate |
| PQBP1 | Monocyte | Innate |
| TEX264 | Monocyte | Innate |
| IKZF1 | Monocyte | Innate |
| AKT3 | Natural killer cell | Innate |
| AXL | Natural killer cell | Innate |
| BST2 | Natural killer cell | Innate |
| CDH2 | Natural killer cell | Innate |
| CRTAM | Natural killer cell | Innate |
| CSF2RA | Natural killer cell | Innate |
| CTSZ | Natural killer cell | Innate |
| CXCL1 | Natural killer cell | Innate |
| CYTH1 | Natural killer cell | Innate |
| DAXX | Natural killer cell | Innate |
| DGKH | Natural killer cell | Innate |
| DLL4 | Natural killer cell | Innate |
| DPYD | Natural killer cell | Innate |
| ERBB3 | Natural killer cell | Innate |
| F11R | Natural killer cell | Innate |
| FAM27A | Natural killer cell | Innate |
| FAM49A | Natural killer cell | Innate |
| FASLG | Natural killer cell | Innate |
| FCGR1A | Natural killer cell | Innate |
| FN1 | Natural killer cell | Innate |
| FSTL1 | Natural killer cell | Innate |
| FUCA1 | Natural killer cell | Innate |
| GBP3 | Natural killer cell | Innate |
| GLS2 | Natural killer cell | Innate |
| GRB2 | Natural killer cell | Innate |
| LST1 | Natural killer cell | Innate |
| BCL2 | Natural killer cell | Innate |
| CDC5L | Natural killer cell | Innate |
| FGF18 | Natural killer cell | Innate |
| FUT5 | Natural killer cell | Innate |
| FZR1 | Natural killer cell | Innate |
| GAGE2 | Natural killer cell | Innate |
| IGFBP5 | Natural killer cell | Innate |
| KANK2 | Natural killer cell | Innate |
| LDB3 | Natural killer cell | Innate |
| BTN2A2 | Natural killer T cell | Innate |
| CD101 | Natural killer T cell | Innate |
| CD109 | Natural killer T cell | Innate |
| CNPY3 | Natural killer T cell | Innate |
| CNPY4 | Natural killer T cell | Innate |
| CREB1 | Natural killer T cell | Innate |
| CRTC2 | Natural killer T cell | Innate |
| CRTC3 | Natural killer T cell | Innate |
| CSF2 | Natural killer T cell | Innate |
| KLRC1 | Natural killer T cell | Innate |
| FUT4 | Natural killer T cell | Innate |
| ICAM2 | Natural killer T cell | Innate |
| IL32 | Natural killer T cell | Innate |
| LAMP2 | Natural killer T cell | Innate |
| LILRB5 | Natural killer T cell | Innate |
| KLRG1 | Natural killer T cell | Innate |
| HSPA4 | Natural killer T cell | Innate |
| HSPB6 | Natural killer T cell | Innate |
| ISM2 | Natural killer T cell | Innate |
| ITIH2 | Natural killer T cell | Innate |
| KDM4C | Natural killer T cell | Innate |
| KIR2DS4 | Natural killer T cell | Innate |
| KIRREL3 | Natural killer T cell | Innate |
| SDCBP | Natural killer T cell | Innate |
| NFATC2IP | Natural killer T cell | Innate |
| MICB | Natural killer T cell | Innate |
| KIR2DL1 | Natural killer T cell | Innate |
| KIR2DL3 | Natural killer T cell | Innate |
| KIR3DL1 | Natural killer T cell | Innate |
| KIR3DL2 | Natural killer T cell | Innate |
| NCR1 | Natural killer T cell | Innate |
| FOSL1 | Natural killer T cell | Innate |
| TSLP | Natural killer T cell | Innate |
| SLC7A7 | Natural killer T cell | Innate |
| SPP1 | Natural killer T cell | Innate |
| TREM2 | Natural killer T cell | Innate |
| UBASH3A | Natural killer T cell | Innate |
| YBX2 | Natural killer T cell | Innate |
| CCDC88A | Natural killer T cell | Innate |
| CLEC1A | Natural killer T cell | Innate |
| THBD | Natural killer T cell | Innate |
| PDPN | Natural killer T cell | Innate |
| VCAM1 | Natural killer T cell | Innate |
| EMR1 | Natural killer T cell | Innate |
| CREB5 | Neutrophil | Innate |
| CDA | Neutrophil | Innate |
| CHST15 | Neutrophil | Innate |
| S100A12 | Neutrophil | Innate |
| APOBEC3A | Neutrophil | Innate |
| CASP5 | Neutrophil | Innate |
| MMP25 | Neutrophil | Innate |
| HAL | Neutrophil | Innate |
| C1orf183 | Neutrophil | Innate |
| FFAR2 | Neutrophil | Innate |
| MAK | Neutrophil | Innate |
| CXCR1 | Neutrophil | Innate |
| STEAP4 | Neutrophil | Innate |
| MGAM | Neutrophil | Innate |
| BTNL8 | Neutrophil | Innate |
| CXCR2 | Neutrophil | Innate |
| TNFRSF10C | Neutrophil | Innate |
| VNN3 | Neutrophil | Innate |
| CBX6 | Plasmacytoid dendritic cell | Innate |
| DAB2 | Plasmacytoid dendritic cell | Innate |
| DDX17 | Plasmacytoid dendritic cell | Innate |
| HIGD1A | Plasmacytoid dendritic cell | Innate |
| IDH3A | Plasmacytoid dendritic cell | Innate |
| IL3RA | Plasmacytoid dendritic cell | Innate |
| MAGED1 | Plasmacytoid dendritic cell | Innate |
| NUCB2 | Plasmacytoid dendritic cell | Innate |
| OFD1 | Plasmacytoid dendritic cell | Innate |
| OGT | Plasmacytoid dendritic cell | Innate |
| PDIA4 | Plasmacytoid dendritic cell | Innate |
| SERTAD2 | Plasmacytoid dendritic cell | Innate |
| SIRPA | Plasmacytoid dendritic cell | Innate |
| TMED2 | Plasmacytoid dendritic cell | Innate |
| ENG | Plasmacytoid dendritic cell | Innate |
| FCAR | Plasmacytoid dendritic cell | Innate |
| IGF1 | Plasmacytoid dendritic cell | Innate |
| ITGA2B | Plasmacytoid dendritic cell | Innate |
| GABARAP | Plasmacytoid dendritic cell | Innate |
| GPX1 | Plasmacytoid dendritic cell | Innate |
| KRT23 | Plasmacytoid dendritic cell | Innate |
| PROK2 | Plasmacytoid dendritic cell | Innate |
| RALB | Plasmacytoid dendritic cell | Innate |
| RETNLB | Plasmacytoid dendritic cell | Innate |
| RNF141 | Plasmacytoid dendritic cell | Innate |
| SEC14L1 | Plasmacytoid dendritic cell | Innate |
| SEPX1 | Plasmacytoid dendritic cell | Innate |
| EMP3 | Plasmacytoid dendritic cell | Innate |
| CD300LF | Plasmacytoid dendritic cell | Innate |
| ABTB1 | Plasmacytoid dendritic cell | Innate |
| KLHL21 | Plasmacytoid dendritic cell | Innate |
| PHRF1 | Plasmacytoid dendritic cell | Innate |

Supplementary Table S2. List of differentially expressed mRNAs

| Gene | HR (95% CI) | P-value |
| --- | --- | --- |
| LY6D | 1.1874(1.1123-1.2677) | 2.61E-07 |
| FAM83A | 1.2295(1.135-1.3319) | 4.08E-07 |
| ANLN | 1.5741(1.3102-1.8911) | 1.27E-06 |
| LAMA3 | 1.5023(1.2726-1.7734) | 1.52E-06 |
| ZNF488 | 1.3765(1.2021-1.5762) | 3.79E-06 |
| MYEOV | 1.3229(1.1745-1.4901) | 4.03E-06 |
| PLAAT2 | 1.2868(1.1551-1.4335) | 4.69E-06 |
| GJB5 | 1.2398(1.1226-1.3693) | 2.22E-05 |
| KRT6A | 1.1324(1.0671-1.2018) | 4.11E-05 |
| PSCA | 1.1469(1.0741-1.2245) | 4.16E-05 |
| S100A2 | 1.1657(1.0801-1.2581) | 8.09E-05 |
| FGFBP1 | 1.1852(1.0886-1.2903) | 8.91E-05 |
| FAM83D | 1.2876(1.1314-1.4654) | 1.28E-04 |
| SDR16C5 | 1.2496(1.1134-1.4024) | 1.53E-04 |
| C19orf33 | 1.2663(1.1204-1.4313) | 1.57E-04 |
| ARL14 | 1.216(1.0966-1.3486) | 2.10E-04 |
| S100P | 1.1825(1.0818-1.2926) | 2.22E-04 |
| SCN11A | 0.6777(0.5499-0.8351) | 2.62E-04 |
| PTK6 | 1.2999(1.1281-1.498) | 2.89E-04 |
| KLK6 | 1.1416(1.0621-1.227) | 3.24E-04 |
| MST1R | 1.3072(1.1258-1.5178) | 4.39E-04 |
| ADGRF1 | 1.2241(1.0924-1.3717) | 4.97E-04 |
| B3GNT3 | 1.4094(1.1616-1.7102) | 5.06E-04 |
| FOXQ1 | 1.3077(1.1239-1.5215) | 5.19E-04 |
| IQANK1 | 1.3979(1.1568-1.6891) | 5.24E-04 |
| MUC1 | 1.2551(1.1035-1.4276) | 5.43E-04 |
| STYK1 | 1.3548(1.14-1.6102) | 5.68E-04 |
| TSPAN1 | 1.3391(1.1338-1.5815) | 5.83E-04 |
| OVOL1 | 1.2454(1.097-1.4139) | 7.02E-04 |
| H2BC5 | 1.3653(1.1389-1.6366) | 7.62E-04 |
| MUCL3 | 1.0977(1.0393-1.1593) | 8.23E-04 |
| IQGAP3 | 1.2982(1.1129-1.5142) | 8.92E-04 |
| H2BC4 | 1.2927(1.1058-1.5113) | 1.28E-03 |
| KCNK1 | 1.3726(1.1315-1.665) | 1.31E-03 |
| GALNT5 | 1.2149(1.0782-1.369) | 1.39E-03 |
| ABHD17C | 1.3267(1.1153-1.5782) | 1.41E-03 |
| DSC3 | 1.1649(1.0599-1.2802) | 1.54E-03 |
| MUC5AC | 1.0967(1.0349-1.1621) | 1.80E-03 |
| ALDH3A1 | 1.1533(1.054-1.2619) | 1.90E-03 |
| MOGAT3 | 1.1506(1.0529-1.2573) | 1.94E-03 |
| PI3 | 1.1425(1.05-1.2431) | 1.97E-03 |
| MROH6 | 1.2393(1.0805-1.4213) | 2.16E-03 |
| MMP12 | 1.1818(1.0621-1.315) | 2.17E-03 |
| AQP5 | 1.1306(1.0453-1.223) | 2.17E-03 |
| SH3TC2 | 1.2913(1.096-1.5215) | 2.25E-03 |
| ABCA12 | 1.1931(1.065-1.3367) | 2.32E-03 |
| NMU | 1.1655(1.055-1.2877) | 2.59E-03 |
| SPDEF | 1.1544(1.0512-1.2678) | 2.66E-03 |
| KRT15 | 1.1904(1.0622-1.3342) | 2.72E-03 |
| PPP1R14D | 1.2068(1.0669-1.365) | 2.79E-03 |
| HTR1D | 1.2309(1.0738-1.411) | 2.86E-03 |
| DNASE1L3 | 0.8248(0.726-0.9371) | 3.09E-03 |
| CFAP65 | 0.8211(0.72-0.9363) | 3.27E-03 |
| H1-2 | 1.2789(1.0821-1.5115) | 3.91E-03 |
| ST6GALNAC1 | 1.1494(1.0442-1.2653) | 4.48E-03 |
| A2ML1 | 1.0979(1.0293-1.1711) | 4.56E-03 |
| CALHM3 | 1.1355(1.0398-1.24) | 4.66E-03 |
| H2BC11 | 1.2513(1.0713-1.4615) | 4.66E-03 |
| NQO1 | 1.242(1.0644-1.4492) | 5.90E-03 |
| MUC5B | 1.1063(1.0287-1.1899) | 6.52E-03 |
| DDC | 0.8843(0.8088-0.9669) | 6.95E-03 |
| TMEM151A | 0.8494(0.7534-0.9576) | 7.65E-03 |
| ABO | 1.1884(1.0466-1.3493) | 7.73E-03 |
| ERN2 | 1.1337(1.0334-1.2438) | 7.91E-03 |
| VSIG2 | 1.145(1.0351-1.2666) | 8.54E-03 |
| CAPN8 | 1.1438(1.0329-1.2666) | 9.81E-03 |
| APOBEC1 | 1.1132(1.026-1.2078) | 9.94E-03 |
| AADAC | 1.1404(1.0314-1.261) | 1.04E-02 |
| CA9 | 1.1076(1.0241-1.1979) | 1.06E-02 |
| TBX15 | 1.1589(1.0347-1.298) | 1.08E-02 |
| VSIG1 | 1.0996(1.021-1.1842) | 1.21E-02 |
| FAM166C | 0.8272(0.7123-0.9606) | 1.29E-02 |
| ABCA13 | 1.1512(1.03-1.2866) | 1.31E-02 |
| GJC2 | 1.1895(1.0371-1.3644) | 1.31E-02 |
| TRIM10 | 1.1799(1.0348-1.3454) | 1.35E-02 |
| MISP | 1.2319(1.0435-1.4544) | 1.38E-02 |
| RAB26 | 0.8482(0.7434-0.9677) | 1.44E-02 |
| SLPI | 1.1699(1.0316-1.3267) | 1.45E-02 |
| MS4A8 | 0.8801(0.7939-0.9757) | 1.52E-02 |
| ZG16B | 1.1381(1.0238-1.2652) | 1.66E-02 |
| OTX1 | 1.2073(1.0305-1.4145) | 1.97E-02 |
| SLC7A11 | 1.1728(1.0249-1.342) | 2.05E-02 |
| CHI3L1 | 1.1444(1.0197-1.2843) | 2.20E-02 |
| FXYD3 | 1.1785(1.0219-1.359) | 2.39E-02 |
| PLA2G10 | 1.1372(1.017-1.2716) | 2.41E-02 |
| CFP | 0.823(0.6935-0.9766) | 2.57E-02 |
| C4BPB | 1.1202(1.0112-1.2408) | 2.97E-02 |
| CETP | 0.7932(0.6399-0.9831) | 3.44E-02 |
| AGR2 | 1.1056(1.0067-1.2141) | 3.57E-02 |
| PLA2G4F | 1.1334(1.0082-1.2742) | 3.60E-02 |
| PPP1R16B | 0.8213(0.6804-0.9914) | 4.04E-02 |
| EPHB3 | 1.1761(1.0061-1.3749) | 4.18E-02 |
| FGFBP2 | 0.8187(0.675-0.993) | 4.23E-02 |
| MATK | 0.8105(0.6601-0.9951) | 4.48E-02 |
| HOXA11 | 1.0913(1.0009-1.1899) | 4.78E-02 |
| LRRC31 | 1.0885(1.0007-1.184) | 4.80E-02 |
| ADAD2 | 0.8342(0.6964-0.9992) | 4.90E-02 |
| PAEP | 1.0761(0.9999-1.1582) | 5.04E-02 |
| GDF7 | 0.8572(0.7345-1.0004) | 5.06E-02 |
| CD36 | 0.8886(0.7885-1.0013) | 5.25E-02 |
| SLC9A4 | 1.0693(0.9989-1.1447) | 5.37E-02 |
| AKR1C4 | 0.8933(0.7964-1.002) | 5.42E-02 |
| XK | 1.1721(0.9967-1.3784) | 5.49E-02 |
| SPIRE2 | 0.853(0.7238-1.0052) | 5.77E-02 |
| IHH | 1.0909(0.9968-1.194) | 5.87E-02 |
| AGR3 | 1.0945(0.9965-1.2021) | 5.91E-02 |
| ADGRG7 | 1.0776(0.9961-1.1658) | 6.25E-02 |
| GIMAP5 | 0.8434(0.7045-1.0097) | 6.36E-02 |
| FIBCD1 | 1.0823(0.9955-1.1767) | 6.38E-02 |
| S100A5 | 1.174(0.9899-1.3924) | 6.52E-02 |
| AMN | 1.104(0.9933-1.2272) | 6.66E-02 |
| HOXA10 | 1.0863(0.9934-1.1879) | 6.96E-02 |
| FAM83E | 1.1204(0.9904-1.2676) | 7.08E-02 |
| SIM2 | 1.1397(0.9887-1.3138) | 7.13E-02 |
| PRAP1 | 1.0662(0.9935-1.1443) | 7.54E-02 |
| NTSR1 | 1.0655(0.993-1.1433) | 7.77E-02 |
| SSTR1 | 0.9159(0.8307-1.0098) | 7.77E-02 |
| SPINK5 | 1.1049(0.9866-1.2375) | 8.44E-02 |
| F5 | 0.9128(0.8212-1.0145) | 9.06E-02 |
| GPRC5D | 1.1491(0.9752-1.354) | 9.69E-02 |
| H2BU1 | 1.1252(0.9751-1.2985) | 1.06E-01 |
| MLPH | 1.1504(0.97-1.3644) | 1.07E-01 |
| KLRK1 | 0.8669(0.7264-1.0346) | 1.13E-01 |
| STAB2 | 0.8965(0.7808-1.0294) | 1.21E-01 |
| ITGAD | 0.88(0.7484-1.0347) | 1.22E-01 |
| DCSTAMP | 1.1141(0.9712-1.278) | 1.23E-01 |
| ALOXE3 | 1.1172(0.9702-1.2866) | 1.24E-01 |
| TFF1 | 1.0487(0.9869-1.1142) | 1.25E-01 |
| PZP | 0.8516(0.6925-1.0472) | 1.28E-01 |
| HEATR9 | 0.8529(0.6949-1.047) | 1.28E-01 |
| ETV4 | 1.1304(0.9652-1.3239) | 1.28E-01 |
| CDHR5 | 1.0772(0.9782-1.1863) | 1.30E-01 |
| HOXC10 | 1.0555(0.982-1.1346) | 1.42E-01 |
| IRF8 | 1.1321(0.9588-1.3368) | 1.43E-01 |
| TBX4 | 1.1029(0.9669-1.2582) | 1.45E-01 |
| H3-5 | 1.2484(0.9259-1.6834) | 1.46E-01 |
| SP5 | 0.897(0.7733-1.0404) | 1.51E-01 |
| ADRA1A | 0.8785(0.7349-1.0502) | 1.55E-01 |
| CCDC187 | 0.9236(0.8269-1.0316) | 1.59E-01 |
| TRIM7 | 1.1111(0.9578-1.289) | 1.64E-01 |
| AC112229.3 | 1.1652(0.9367-1.4494) | 1.70E-01 |
| AKR7A3 | 1.0889(0.9635-1.2306) | 1.72E-01 |
| DMBT1 | 1.0423(0.9814-1.107) | 1.78E-01 |
| SLC9A2 | 1.0661(0.9713-1.1702) | 1.78E-01 |
| KLRD1 | 1.1253(0.9473-1.3367) | 1.79E-01 |
| BAIAP2L2 | 1.0918(0.9601-1.2416) | 1.80E-01 |
| CDH16 | 1.0684(0.9697-1.1772) | 1.81E-01 |
| MOGAT2 | 1.0586(0.9706-1.1545) | 1.99E-01 |
| CAPN9 | 1.0536(0.9699-1.1445) | 2.17E-01 |
| TMEM45B | 1.096(0.9459-1.27) | 2.22E-01 |
| MIA | 1.0521(0.9695-1.1417) | 2.24E-01 |
| GPR55 | 0.9099(0.7781-1.064) | 2.37E-01 |
| ALG1L | 1.0719(0.9487-1.2111) | 2.65E-01 |
| LILRB5 | 0.9246(0.8048-1.0624) | 2.69E-01 |
| CLRN3 | 1.0572(0.9572-1.1678) | 2.72E-01 |
| KLRF1 | 1.1212(0.9122-1.3781) | 2.77E-01 |
| HBA2 | 0.9466(0.856-1.0469) | 2.86E-01 |
| CLCA4 | 1.0575(0.9533-1.1731) | 2.91E-01 |
| HK3 | 1.0777(0.9351-1.2419) | 3.02E-01 |
| P2RY13 | 0.9333(0.8175-1.0655) | 3.07E-01 |
| ADGRE1 | 0.9239(0.7901-1.0804) | 3.22E-01 |
| ATP2C2 | 1.0742(0.9316-1.2386) | 3.25E-01 |
| AQP6 | 1.0584(0.9417-1.1895) | 3.41E-01 |
| IL4I1 | 1.08(0.9209-1.2666) | 3.44E-01 |
| SPATC1 | 0.9233(0.7726-1.1034) | 3.80E-01 |
| BTNL8 | 1.0349(0.9565-1.1197) | 3.93E-01 |
| TUBAL3 | 1.0537(0.9333-1.1896) | 3.98E-01 |
| RHBDL1 | 0.9359(0.8021-1.092) | 4.00E-01 |
| ENTPD8 | 1.0521(0.9341-1.1849) | 4.03E-01 |
| SLC4A11 | 1.0604(0.9225-1.2188) | 4.09E-01 |
| PPP2R2C | 1.0401(0.9441-1.1459) | 4.26E-01 |
| CD300LB | 0.9375(0.7944-1.1063) | 4.45E-01 |
| CD180 | 1.0534(0.9181-1.2087) | 4.58E-01 |
| MUC2 | 1.0178(0.9702-1.0677) | 4.70E-01 |
| MYO1A | 1.032(0.947-1.1246) | 4.72E-01 |
| CD72 | 1.0641(0.8972-1.262) | 4.75E-01 |
| PRF1 | 1.0751(0.8798-1.3138) | 4.79E-01 |
| SCIMP | 0.9458(0.8094-1.1054) | 4.84E-01 |
| TNNT2 | 1.0401(0.9312-1.1618) | 4.86E-01 |
| CHRNA5 | 1.0633(0.8921-1.2674) | 4.93E-01 |
| HAPLN1 | 1.0353(0.9336-1.148) | 5.11E-01 |
| IL18RAP | 0.9424(0.7887-1.1261) | 5.14E-01 |
| SOSTDC1 | 1.03(0.9387-1.1302) | 5.33E-01 |
| POU2F2 | 0.9422(0.7814-1.1361) | 5.33E-01 |
| H2BC21 | 1.0609(0.8792-1.2803) | 5.37E-01 |
| CLECL1 | 0.9549(0.8237-1.1071) | 5.41E-01 |
| CNTD2 | 0.9637(0.8546-1.0868) | 5.47E-01 |
| LEFTY2 | 0.9514(0.8085-1.1195) | 5.48E-01 |
| SLC28A2 | 0.9688(0.8735-1.0745) | 5.49E-01 |
| MUC13 | 1.0291(0.9359-1.1315) | 5.54E-01 |
| CCL2 | 0.963(0.8458-1.0966) | 5.70E-01 |
| LILRA4 | 0.9713(0.8709-1.0833) | 6.01E-01 |
| LILRA1 | 0.959(0.8195-1.1222) | 6.02E-01 |
| TMEM82 | 1.0311(0.917-1.1596) | 6.09E-01 |
| SPACA4 | 1.0329(0.9088-1.1739) | 6.20E-01 |
| CNNM1 | 0.9687(0.8529-1.1003) | 6.25E-01 |
| PLEK | 1.037(0.8959-1.2002) | 6.26E-01 |
| ANKS4B | 0.9752(0.8784-1.0826) | 6.37E-01 |
| JPH1 | 1.0417(0.871-1.2459) | 6.54E-01 |
| CD52 | 1.0326(0.8971-1.1886) | 6.55E-01 |
| FGD2 | 1.0427(0.8671-1.2539) | 6.57E-01 |
| EOMES | 0.9716(0.8547-1.1044) | 6.59E-01 |
| MAB21L4 | 1.0301(0.8949-1.1856) | 6.80E-01 |
| VSTM2L | 1.02(0.9221-1.1284) | 7.00E-01 |
| RIBC2 | 0.9703(0.8217-1.1458) | 7.22E-01 |
| CYP3A4 | 1.0162(0.9244-1.1171) | 7.40E-01 |
| KMO | 0.9708(0.8072-1.1677) | 7.53E-01 |
| BTK | 1.0241(0.8819-1.1893) | 7.55E-01 |
| FABP6 | 0.9863(0.9044-1.0757) | 7.56E-01 |
| FGR | 1.0263(0.8659-1.2164) | 7.65E-01 |
| NKG7 | 1.0261(0.8617-1.222) | 7.72E-01 |
| ARHGAP25 | 0.9747(0.8064-1.1781) | 7.91E-01 |
| PRSS33 | 1.0103(0.9308-1.0967) | 8.06E-01 |
| MCOLN2 | 0.9821(0.8458-1.1404) | 8.13E-01 |
| WDFY4 | 0.9856(0.8703-1.1162) | 8.19E-01 |
| SASH3 | 0.9825(0.8422-1.1461) | 8.22E-01 |
| PARVG | 0.9808(0.8253-1.1655) | 8.26E-01 |
| NCF1 | 0.9839(0.8469-1.1431) | 8.32E-01 |
| PTGDR | 0.982(0.8251-1.1687) | 8.38E-01 |
| HR | 1.0178(0.8591-1.2057) | 8.39E-01 |
| CKMT1B | 0.985(0.847-1.1456) | 8.45E-01 |
| HS3ST5 | 1.0111(0.8954-1.1417) | 8.59E-01 |
| TOX3 | 1.0131(0.8722-1.1769) | 8.64E-01 |
| CKMT1A | 0.9871(0.84-1.1599) | 8.74E-01 |
| LILRB1 | 0.9881(0.8382-1.1649) | 8.87E-01 |
| CD244 | 1.0106(0.8493-1.2026) | 9.05E-01 |
| SLC27A2 | 1.0078(0.8763-1.159) | 9.14E-01 |
| ELFN2 | 0.997(0.8802-1.1294) | 9.63E-01 |
| FOXA3 | 0.9974(0.8687-1.1451) | 9.70E-01 |
| SIGLEC11 | 0.9974(0.8624-1.1537) | 9.73E-01 |
| ITGAL | 0.9983(0.8705-1.1449) | 9.80E-01 |
| FCRL6 | 1.0008(0.8481-1.181) | 9.93E-01 |
| TBX21 | 1.0007(0.847-1.1824) | 9.93E-01 |
| LRRC66 | 1.0001(0.8736-1.1449) | 9.99E-01 |

Supplementary Table S3.The results of univariate Cox regression analysis

| Gene | HR (95% CI) | P-value |
| --- | --- | --- |
| LY6D | 1.1874(1.1123-1.2677) | 2.61E-07 |
| FAM83A | 1.2295(1.135-1.3319) | 4.08E-07 |
| ANLN | 1.5741(1.3102-1.8911) | 1.27E-06 |
| LAMA3 | 1.5023(1.2726-1.7734) | 1.52E-06 |
| ZNF488 | 1.3765(1.2021-1.5762) | 3.79E-06 |
| MYEOV | 1.3229(1.1745-1.4901) | 4.03E-06 |
| PLAAT2 | 1.2868(1.1551-1.4335) | 4.69E-06 |
| GJB5 | 1.2398(1.1226-1.3693) | 2.22E-05 |
| KRT6A | 1.1324(1.0671-1.2018) | 4.11E-05 |
| PSCA | 1.1469(1.0741-1.2245) | 4.16E-05 |
| S100A2 | 1.1657(1.0801-1.2581) | 8.09E-05 |
| FGFBP1 | 1.1852(1.0886-1.2903) | 8.91E-05 |
| FAM83D | 1.2876(1.1314-1.4654) | 1.28E-04 |
| SDR16C5 | 1.2496(1.1134-1.4024) | 1.53E-04 |
| C19orf33 | 1.2663(1.1204-1.4313) | 1.57E-04 |
| ARL14 | 1.216(1.0966-1.3486) | 2.10E-04 |
| S100P | 1.1825(1.0818-1.2926) | 2.22E-04 |
| SCN11A | 0.6777(0.5499-0.8351) | 2.62E-04 |
| PTK6 | 1.2999(1.1281-1.498) | 2.89E-04 |
| KLK6 | 1.1416(1.0621-1.227) | 3.24E-04 |
| MST1R | 1.3072(1.1258-1.5178) | 4.39E-04 |
| ADGRF1 | 1.2241(1.0924-1.3717) | 4.97E-04 |
| B3GNT3 | 1.4094(1.1616-1.7102) | 5.06E-04 |
| FOXQ1 | 1.3077(1.1239-1.5215) | 5.19E-04 |
| IQANK1 | 1.3979(1.1568-1.6891) | 5.24E-04 |
| MUC1 | 1.2551(1.1035-1.4276) | 5.43E-04 |
| STYK1 | 1.3548(1.14-1.6102) | 5.68E-04 |
| TSPAN1 | 1.3391(1.1338-1.5815) | 5.83E-04 |
| OVOL1 | 1.2454(1.097-1.4139) | 7.02E-04 |
| H2BC5 | 1.3653(1.1389-1.6366) | 7.62E-04 |
| MUCL3 | 1.0977(1.0393-1.1593) | 8.23E-04 |
| IQGAP3 | 1.2982(1.1129-1.5142) | 8.92E-04 |
| H2BC4 | 1.2927(1.1058-1.5113) | 1.28E-03 |
| KCNK1 | 1.3726(1.1315-1.665) | 1.31E-03 |
| GALNT5 | 1.2149(1.0782-1.369) | 1.39E-03 |
| ABHD17C | 1.3267(1.1153-1.5782) | 1.41E-03 |
| DSC3 | 1.1649(1.0599-1.2802) | 1.54E-03 |
| MUC5AC | 1.0967(1.0349-1.1621) | 1.80E-03 |
| ALDH3A1 | 1.1533(1.054-1.2619) | 1.90E-03 |
| MOGAT3 | 1.1506(1.0529-1.2573) | 1.94E-03 |
| PI3 | 1.1425(1.05-1.2431) | 1.97E-03 |
| MROH6 | 1.2393(1.0805-1.4213) | 2.16E-03 |
| MMP12 | 1.1818(1.0621-1.315) | 2.17E-03 |
| AQP5 | 1.1306(1.0453-1.223) | 2.17E-03 |
| SH3TC2 | 1.2913(1.096-1.5215) | 2.25E-03 |
| ABCA12 | 1.1931(1.065-1.3367) | 2.32E-03 |
| NMU | 1.1655(1.055-1.2877) | 2.59E-03 |
| SPDEF | 1.1544(1.0512-1.2678) | 2.66E-03 |
| KRT15 | 1.1904(1.0622-1.3342) | 2.72E-03 |
| PPP1R14D | 1.2068(1.0669-1.365) | 2.79E-03 |
| HTR1D | 1.2309(1.0738-1.411) | 2.86E-03 |
| DNASE1L3 | 0.8248(0.726-0.9371) | 3.09E-03 |
| CFAP65 | 0.8211(0.72-0.9363) | 3.27E-03 |
| H1-2 | 1.2789(1.0821-1.5115) | 3.91E-03 |
| ST6GALNAC1 | 1.1494(1.0442-1.2653) | 4.48E-03 |
| A2ML1 | 1.0979(1.0293-1.1711) | 4.56E-03 |
| CALHM3 | 1.1355(1.0398-1.24) | 4.66E-03 |
| H2BC11 | 1.2513(1.0713-1.4615) | 4.66E-03 |
| NQO1 | 1.242(1.0644-1.4492) | 5.90E-03 |
| MUC5B | 1.1063(1.0287-1.1899) | 6.52E-03 |
| DDC | 0.8843(0.8088-0.9669) | 6.95E-03 |
| TMEM151A | 0.8494(0.7534-0.9576) | 7.65E-03 |
| ABO | 1.1884(1.0466-1.3493) | 7.73E-03 |
| ERN2 | 1.1337(1.0334-1.2438) | 7.91E-03 |
| VSIG2 | 1.145(1.0351-1.2666) | 8.54E-03 |
| CAPN8 | 1.1438(1.0329-1.2666) | 9.81E-03 |
| APOBEC1 | 1.1132(1.026-1.2078) | 9.94E-03 |
